# Rhizosphere bacteriome structure and functions

**DOI:** 10.1038/s41467-022-28448-9

**Published:** 2022-02-11

**Authors:** Ning Ling, Tingting Wang, Yakov Kuzyakov

**Affiliations:** 1 Centre for Grassland Microbiome, State Key Laboratory of Grassland Agro-ecosystems, College of Pastoral Agricultural Science and Technology, Lanzhou University, Lanzhou, 730020 Gansu China; 2 Jiangsu Provincial Key Lab for Organic Solid Waste Utilization, Jiangsu Collaborative Innovation Center for Solid Organic Waste Resource Utilization, Nanjing Agricultural University, Nanjing, 210095 China; 3 Department of Soil Science of Temperate Ecosystems, Department of Agricultural Soil Science, University of Goettingen, 37077 Göttingen, Germany; 4 Peoples Friendship University of Russia (RUDN University), 117198 Moscow, Russia

**Keywords:** Agroecology, Microbial ecology, Agroecology

## Abstract

Microbial composition and functions in the rhizosphere—an important microbial hotspot—are among the most fascinating yet elusive topics in microbial ecology. We used 557 pairs of published 16S rDNA amplicon sequences from the bulk soils and rhizosphere in different ecosystems around the world to generalize bacterial characteristics with respect to community diversity, composition, and functions. The rhizosphere selects microorganisms from bulk soil to function as a seed bank, reducing microbial diversity. The rhizosphere is enriched in Bacteroidetes, Proteobacteria, and other copiotrophs. Highly modular but unstable bacterial networks in the rhizosphere (common for *r*-strategists) reflect the interactions and adaptations of microorganisms to dynamic conditions. Dormancy strategies in the rhizosphere are dominated by toxin–antitoxin systems, while sporulation is common in bulk soils. Functional predictions showed that genes involved in organic compound conversion, nitrogen fixation, and denitrification were strongly enriched in the rhizosphere (11–182%), while genes involved in nitrification were strongly depleted.

## Abstract (web summary)

Understanding soil microbiota dynamics is key the development of soil-based sustainable agriculture and conservation strategies. This meta-analysis shows that bulk soil functions as a seed bank for the rhizosphere, which encompasses a rich microbiota adapted to dynamic conditions in hotpots.

## Introduction

The rhizosphere is home to a rich diversity of microorganisms, many of which benefit plants by suppressing pathogenic invasions and helping to acquire nutrients from the soil^1,2^. Understanding the taxonomic and functional components of the rhizosphere microbiome and how they differ from those of the bulk soil microbiome (here: soil without direct root effects) is crucial to manipulate them for sustainable ecosystem functioning. Recent advances in sequencing have enabled significant progress in the elucidation of the rhizosphere microbiomes of various plants. The diversity and composition of the rhizosphere bacterial community is a function of both plant species and soil properties^3–5^. Although plant species or even species genotypes tend to assemble relatively distinct rhizobacterial communities^6–8^, these communities can be largely similar even in different environments across geographical regions^9,10^. Plants exert selective effects on rhizobacterial assemblages in bulk soil pool to acquire specific functional traits needed for plant fitness^8,11,12^. As a result, the rhizosphere microbiome greatly expands the functional repertoire of the plant^13^. Despite the specific nutrient requirements of plants, disease control mechanisms, and edaphic habitats, rhizosphere environments (with excess available carbon) provide broadly similar conditions for microbial life. All plants are mineral resource-limited organisms and are often affected by pathogens. To overcome these limitations, plants form rhizo-assemblages independent of their host phylogeny. Thus, all plants can exert general selective effects directed toward nutrient acquisition or pathogen suppression, regardless of their geographic origin or recent location. These general patterns in the rhizosphere and bulk soils with respect to the taxonomic and functional profiles of bacterial communities remain largely unexplored. However, this information is critical to understand and manage microbial functions in ecosystems to support future plant growth in rapidly changing environment.

Recently, high-throughput sequencing of culture-independent marker genes (typically, 16S rRNA in the case of bacteria), has greatly expanded the repertoire of microorganisms living in soils^14^, and many studies have characterized root-associated microbial communities^9^. Since the raw data from most studies must be deposited in a public gene bank, this has resulted in a huge and extensive rhizosphere sequencing data set. These high-resolution nucleic acid-based molecular techniques provide excellent insights into specific microbiome members in soil habitats. Research priorities for harnessing the rhizosphere microbiome for sustainable ecosystems development include elucidating the functional mechanisms that mediate plant-microbiome interactions and defining the core of the plant microbiome^15,16^. The methodological advances made in these priority research areas have provided a vast amount of data for integrative analysis and subsequent synthesis. This has paved the way for investigating the general principles of rhizosphere microbiome selection from bulk soils. The rhizosphere contains numerous niches for the growth and proliferation of a phylogenetically diverse array of microorganisms, including bacteria, archaea, fungi, protists, nematodes, and viruses, but bacteria and, to a lesser extent, fungi are the most dominant forms and are fairly well-studied compared to other members of the community. Thus, we are attempting to infer the composition of bacterial communities in the rhizosphere and bulk soil, determine the rhizosphere bacteriome properties common to a wide range of plants and environmental conditions, and bridge the gap between general rhizobacterial assemblages and functions associated with community-wide dormancy capacity, heterotrophic strategies, and individual nutrient cycling processes.

Here, we collected 16S rRNA amplicon-based sequencing data from all available gene banks to characterize the general bacteriome and synthetically analyzed the data using state-of-the-art bioinformatics methods. Bulk soil serves as a microbial reservoir for the rhizo-microbiome^17^ and is home to considerable microbial diversity that is explicitly shaped by the environmental factors of each microhabitat^18,19^. We, therefore, hypothesized that (i) rhizobacterial populations are recruited primarily from the corresponding bulk soil, but are preselected by excess released root carbon, so that bacterial diversity is generally lower in the rhizosphere and bacterial networks are less stable. Compared to bulk soils, the rhizosphere can fuel its microbiota by providing abundant and readily available energy and carbon sources^20,21^, but microorganisms in root-free soil are always carbon limited^22^. We therefore further hypothesized that (ii) the rhizosphere is home to more abundant copiotrophic bacteria than the bulk soil. To this end, we evaluated the community weighted mean 16S rRNA gene copies (rRNA operons) because copiotrophs are assumed to have more rRNA operons than oligotrophs^23,24^. Since all nutrients flow from the bulk soil to the roots, we further hypothesized that (iii) the functional capacity involved in the carbon and nitrogen transformation would be greater in the rhizosphere. Plants deposit a significant proportion of their photosynthates in the rhizosphere as rhizodeposits and root debris. The rhizodeposits, including amino acids, carboxylic acids, sugars and polymeric carbohydrates such as cellulose and hemicellulose^25^, are not only critical carbon and energy sources for rhizobacteria but also key attractants for plant pathogens. We finally hypothesized that (iv) functions related to lignocellulose degradation and phytopathogens are overexpressed in the rhizosphere due to the accumulation of root litter and the attraction of pathogens to live plants. In this work, we analyzed and synthesized a very broad range of taxonomic and functional features of the bacteriome in the rhizosphere compared to bulk soil, and generalized these compositional changes to other factors such as plant species, geographic environment, and soil properties. This provided general principles for the selection of microorganisms around living roots and laid the foundation for harnessing the power of the microbiome for sustainable terrestrial ecosystem functioning.

## Results

### Bacterial diversity and community composition in the rhizosphere and bulk soils

**Fig. 1:**
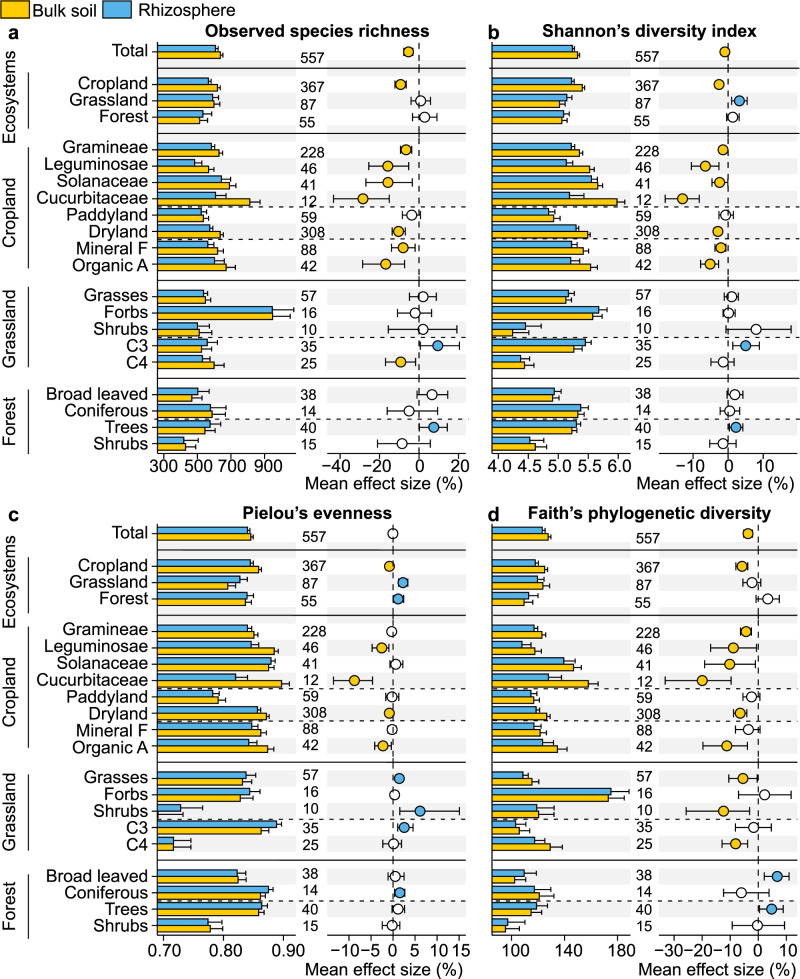
Diversity of bacterial communities: the rhizosphere versus bulk soil. **a** observed species richness; **b** Shannon’s diversity index; **c** Pielou’s evenness; and **d** Faith’s phylogenetic diversity. Bar charts reflect mean value and standard errors for each category. The error bars on the columns represent the standard errors (SE). All dots represent the percentage change in effect size between rhizosphere and bulk soil bacterial diversity at 95% confidence intervals (CIs). Mean values <0 indicate greater diversity in the bulk soil bacterial community (yellow dots; depletion in the rhizosphere), while mean values >0 reflect significantly greater diversity in the rhizosphere bacterial community (blue dots). The intersection of the error bars and the zero line indicates that there is no significant difference between the bacterial communities in the rhizosphere and in the bulk soils (open dots). Sample size is showed by number of data pairs for each group. Mineral F and Organic A indicate the types of fertilization, which are mineral-only fertilization and organic amendments, respectively. Source data are provided as a Source Data file.

The dataset was collected from public databases containing published papers across continents. The synthesis of bacterial sequences from terrestrial ecosystems—bulk versus rhizosphere soil pairs (Supplementary Fig. 1)—provided a generalized pattern of alpha-diversity. Across all studies, bacterial alpha-diversity is depleted in the rhizosphere compared to the bulk soil in terms of observed species richness (−5.3%), Shannon’s diversity index (−0.9%), and Faith’s phylogenetic diversity (−3.7%) (Fig. 1). The funnel plot with the trim-and-fill method confirmed that there was little bias on alpha-diversity (Supplementary Table 1 and Supplementary Fig. 2). Studies of specific ecosystems showed a consistent decrease in alpha-diversity in the rhizosphere (−0.8% ~ −9.3%) in cultivated land, while no significant differences in observed species richness and Faith’s phylogenetic diversity were found between bulk soil and the rhizosphere in grassland and forest ecosystems. In agricultural bulk soils planted with Gramineae, Leguminosae, Solanaceae, and Cucurbitaceae, almost all alpha-diversity indices were significantly higher than in the rhizosphere. However, under mineral fertilization, the Pielou’s evenness and Faith’s phylogenetic diversity indices lost their significance. This indicates that mineral-only fertilization disrupts biodiversity in bulk soils. Contrary to our expectation, the alpha diversity in the rice rhizosphere was similar to that of the bulk soil (Fig. 1). Consequently, the increase in microbial diversity under high redox potential, especially the redox variation around rice roots, was offset by a decrease in diversity due to excess available carbon and its rapid diffusion from the root surface.

When grassland ecosystems were grouped by plant type and traits, rhizosphere bacteria were generally enriched in C3 grasses, whereas they were commonly depleted in C4 grasses. Despite the fact that the plant groups can shape bacterial alpha-diversity in the rhizosphere, Faith’s phylogenetic diversity was higher in bulk soil under grasses and shrubs in grassland ecosystems. In forest ecosystems, bacterial diversity, including the observed species richness, Shannon’s diversity index, and Faith’s phylogenetic diversity, tended to be greater in trees’ rhizosphere than in the bulk soils, but the alpha-diversity in the rhizosphere of shrubs was similar to that in the bulk soils.

We considered the potential effects of sequencing platform, target regions of primer pairs, and experimental management (field or greenhouse) to further dissect how these factors altered the effect size (Supplementary Fig. 3). No significant differences were found between the field and greenhouse experiments. In terms of sequencing platforms, the majority of the sequencing methods used the Illumina platform, and although a small number of sequencing methods used the Ion S5 platform, the overall effect size remained the same. There was negligible variation in effect size by target region, confirming that the major variations mainly presented among the ecosystem types (Supplementary Fig. 3).

**Fig. 2:**
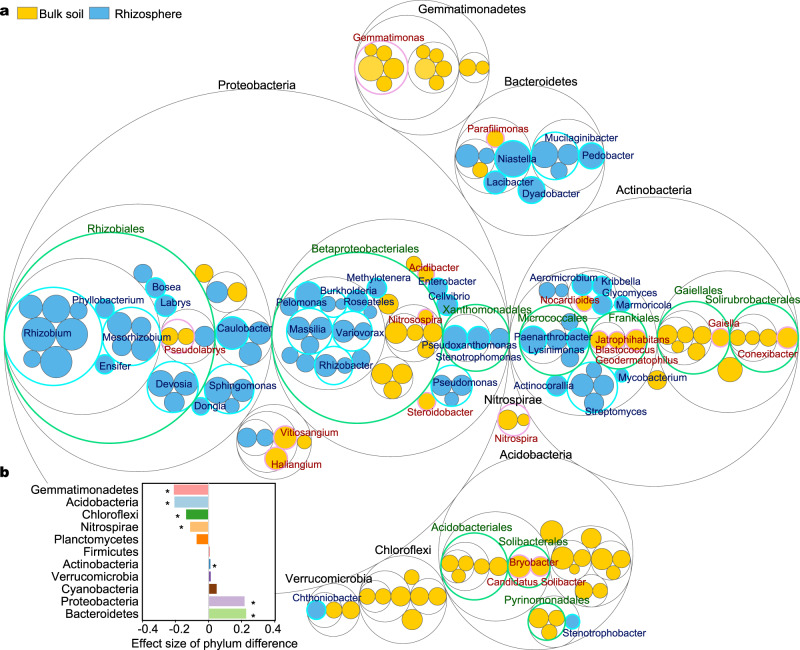
Differences in relative abundance of bacterial taxa between the rhizosphere and bulk soil. **a** Difference at the amplicon sequence variants (ASV) level. Blue and yellow bubbles represent ASVs with significant enrichment (FDR < 0.05) in rhizosphere and bulk soil, respectively, based on statistical analysis with ALDEx2. Bubble sizes represent the ALDEx2 effect size between the relative abundance of ASV in the rhizosphere and in the bulk soils. The largest circles in black represent the phylum level, and the innermost circles in green represent the order level. All ASVs that can be annotated at genus level were marked next to the corresponding bubbles. **b** Differences at phylum level. Positive values indicate higher relative abundance of the phylum in rhizosphere, while negative values indicate higher relative abundance of the phylum in the bulk soil. The differences were statistically analyzed using ALDEx2. An asterisk (*) indicates a significant difference with FDR < 0.05 based on statistical analysis with ALDEx2. Source data are provided as a Source data file.

The principal coordinates analysis with Bray-Curtis dissimilarity and the permutational multivariate analysis of variance (PERMANOVA) showed a significantly difference (*p* = 0.01; Supplementary Fig. 4) between the bacterial community compositions in bulk and rhizosphere soils. The bacterial phyla—Bacteroidetes and Proteobacteria—were enriched in the rhizosphere of overall samples, whereas Chloroflexi, Acidobacteria, Gemmatimonadetes, and Nitrospirae were generally enriched in the bulk soil (Fig. 2 and Supplementary Fig. 5). Although the relative abundance of Proteobacteria was higher in the rhizosphere, the genera Haliangium, Pseudolabrys, Acidibacter, and Nitrosospira, belonging to the phylum Proteobacteria, were much richer in the bulk soil (Fig. 2 and Supplementary Fig. 6). The relative abundance of the phylum Actinobacteria was also much higher in the rhizosphere of most plants, while some of its genera such as Gaiella, Blastococcus, Nocardioides, and Conexibacter were mainly localized in the bulk soil.

When the dataset was divided by plant group, Firmicutes were abundant in rhizosphere of Gramineae crops, while this phylum was depleted in several plant groups such as Solanaceae and Cucurbitaceae crops and grasses (Supplementary Fig. 5). Cyanobacteria were enriched in the rhizosphere of the Cucurbitaceae crops and grasses. Planctomycetes were similar in the bulk soil and the rhizosphere of grasses, forbs, and trees, but reduced in the rhizosphere of crops. The Rhizobium and Mesorhizobium genus had higher relative abundance in the rhizosphere of most plants, especially Gramineae, Leguminosae, Cucurbitaceae, and grasses (Supplementary Fig. 7). However, some bacterial genera were enriched only in specific plant groups. For instance, Burkholderia and Variovorax were higher only in the rhizosphere of Leguminosae, and the genera Pedobacter and Aeromicrobium were enriched only in the rhizosphere of Gramineae. Bacterial enrichment under forbs and trees differed from that in the agro-ecosystems.

### Co-occurrence patterns in the rhizosphere and bulk soils

**Fig. 3:**
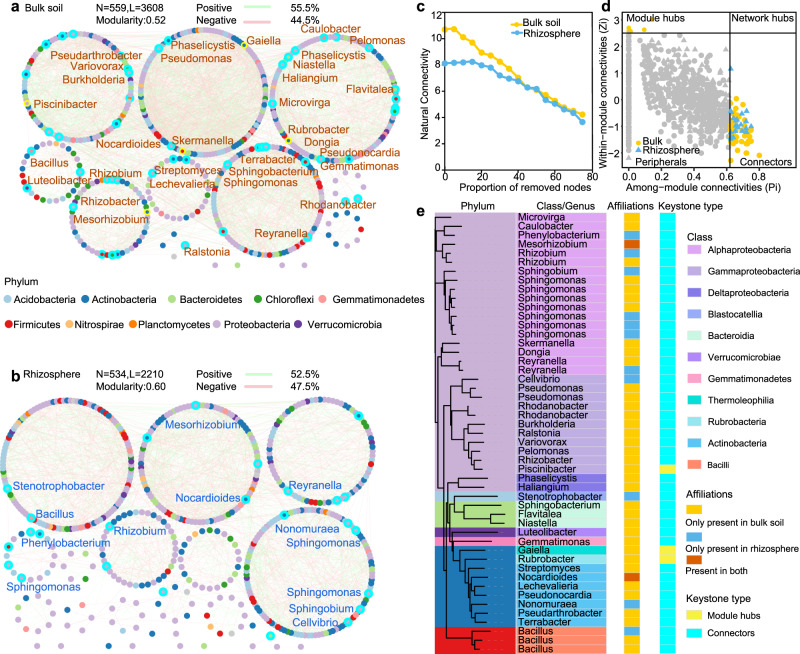
Global co-occurrence networks of bacterial amplicon sequence variants (ASVs) in the rhizosphere and in bulk soil. **a** a co-occurrence in the global bulk soil; **b**, a co-occurrence in the global rhizosphere. Colors of nodes indicate the different major phyla. **c** Robustness of global bacterial networks in bulk soil (yellow dots) and the rhizosphere (blue dots) (bulk soils *n* = 1759 vs. rhizosphere *n* = 2182); **d** classification of nodes to identify the keystone ASVs in the networks. **e** Phylogenetic tree of keystone ASVs in the networks. The taxonomy of keystone ASVs is also labeled at the genus level in yellow in the global bulk soil network (**a**) and in blue in the global rhizosphere network (**b**). Source data are provided as a Source data file.

In order to characterize the general co-occurrence pattern of bacteriome networks in bulk soil and rhizosphere, all plants were pooled to construct networks which were called global networks (i.e., global bulk soil network and global rhizosphere network). Multiple network topological metrics consistently showed that the global networks of bacterial communities in the bulk soil (Fig. 3a) differed from those in the rhizosphere (Fig. 3b). The global network in the bulk soil has 559 nodes and 3608 edges, whereas the global network in the rhizosphere has 534 nodes and 2210 edges (Fig. 3, Supplementary Table 2). The global rhizosphere network features more modules (Fig. 3, Supplementary Table 2), but it is much less robust and stable (Fig. 3c). This is evidenced by the fact that removing nodes at any level always results in a low degree of natural connectivity (Fig. 3c). In both global networks, Proteobacteria and Actinobacteria were dominant in keystone nodes (Fig. 3d, e). However, the amplicon sequence variants (ASVs) that highly connect the nodes among modules were different (Fig. 3e): in the global rhizosphere network, a few ASVs were found to act as connectors (i.e., nodes that highly connect modules), and they mainly belonged to the Alphaproteobacteria, Gammaproteobacteria, Blastocatellia, Actinobacteria, and Bacilli classes. On the other hand, module hubs (highly connected nodes within modules) were only detected in the global bulk soil network, where the genera *Piscinibacter* sp., *Gaiella* sp., and *Rubrobacter* sp. served that function. The keystone ASVs classified as *Mesorhizobium* sp. and *Nocardioides* sp. were found to act as connectors in both global networks.

To further characterize the influence of plants on bacteriome networks, we assessed co-occurrence patterns based on plant groups (Supplementary Fig. 8), and defined these networks as local networks. Various plant groups had similarly strong effects on network complexity. Bacterial network modularity trended to increase from the bulk soil towards the rhizosphere (Supplementary Fig. 8a, b). The connector species in the network depended on the plant type (Supplementary Fig. 8d), and the topological role of keystone microbial species depended on plant traits. The number of species serving as inter-module connectors differed between the global network (i.e., a network of all plants) and the local network (i.e., a network of specific groups of plants) (Fig. 3 and Supplementary Fig. 8). This result further indicates that the keystone taxa in the organizing network topology retain their context dependence, and that some organisms may play the same functional role in maintaining network stability.

### Dormancy potential and heterotrophic strategies at the bacterial community level

Bacterial community characteristics related to dormancy potential and heterotrophic strategies have important implications for the restoration and maintenance of ecosystem functioning underpinned by resource availability. These community-level characteristics reveal resource-based differences in bulk and rhizosphere soil patterns. The low bias of the response ratio data was confirmed by the funnel plot with Egger’s regression test and the trim-and-fill analysis (Supplementary Fig. 9 and Supplementary Table 1).

**Fig. 4:**
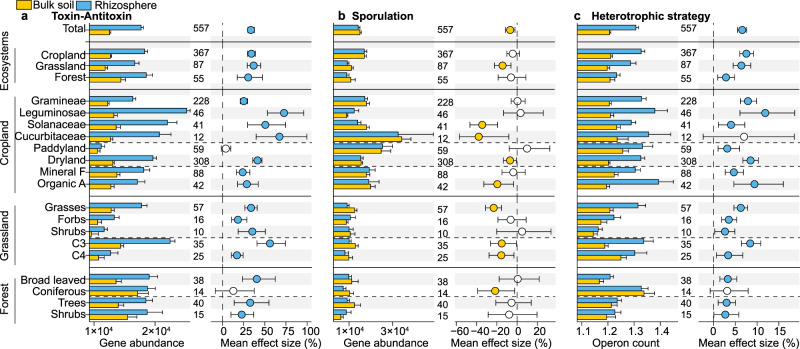
Dormancy potentials and heterotrophic strategies of bacterial communities in the rhizosphere and bulk soils. **a** Differences in the abundances of toxin–antitoxin systems genes between communities in the rhizosphere and bulk soils; **b** differences in the sporulation factor abundances between communities in the rhizosphere and bulk soils; **c** differences in the weighted mean ribosomal operon copy numbers between communities in the rhizosphere and bulk soils. Bar charts reflect mean value and standard errors for each category. The error bars on the columns represent the standard errors (SE). Dots represent the percentage change in effect size between bacterial communities in the rhizosphere and bulk soils with 95% confidence intervals (CIs). Mean values <0 indicate a higher dormancy potential or heterotrophic strategy in bulk soil (yellow dots: depletion in the rhizosphere), while mean values >0 reflect a higher dormancy potential or heterotrophic strategy in the rhizosphere (blue dots). The intersection of the error bars and the zero line indicates that there is no significant difference between bacterial communities in the rhizosphere and bulk soils (open dots). Sample size is showed by number of data pairs for each group. Mineral F and Organic A indicate the types of fertilization, which are mineral-only fertilization and organic amendments, respectively. Source data are provided as a Source data file.

Dormancy potential, characterized by genes involved in toxin–antitoxin and sporulation, was evaluated to clarify bacterial community-aggregated dormancy strategies in the rhizosphere and bulk soil. We found that the toxin–antitoxin functional potential was 33% higher in the former (Fig. 4a) and sporulation functional potential was 7% higher in the latter (Fig. 4b), while the magnitude of the effect (bulk soil vs. rhizosphere soil) was influenced by ecosystem type and plant group. In general, bacteria inhabiting the rhizosphere have distinctly different dominant strategies for entering dormancy compared to bacteria living in bulk soils. Interestingly, in paddy soils, conditions eliminated the bacterial community with a well-developed toxin–antitoxin system in the rhizosphere, indicating that land use strongly influences bacterial dormancy potential.

Heterotrophic strategies for community-level aggregated traits were characterized by weighted mean rRNA operon copy number. Bacteria with higher rRNA operon counts are able to maintain higher maximal growth rates and respond faster to resources. The rhizosphere had 6.6% more rRNA operon counts (Fig. 4c), indicating that more fast-growing bacteria (*r*-strategists) preferentially colonize the rhizosphere than the bulk soil. Heterotrophic strategies at the bacterial community level were also influenced by the land use regime: the effect of rhizosphere on heterotrophic strategies was weaker in paddy fields than in dryland soils, with the greatest effect in the Leguminosae rhizosphere, which was 12% higher than in bulk soils.

### Functional signatures in the rhizosphere and bulk soils

**Fig. 5:**
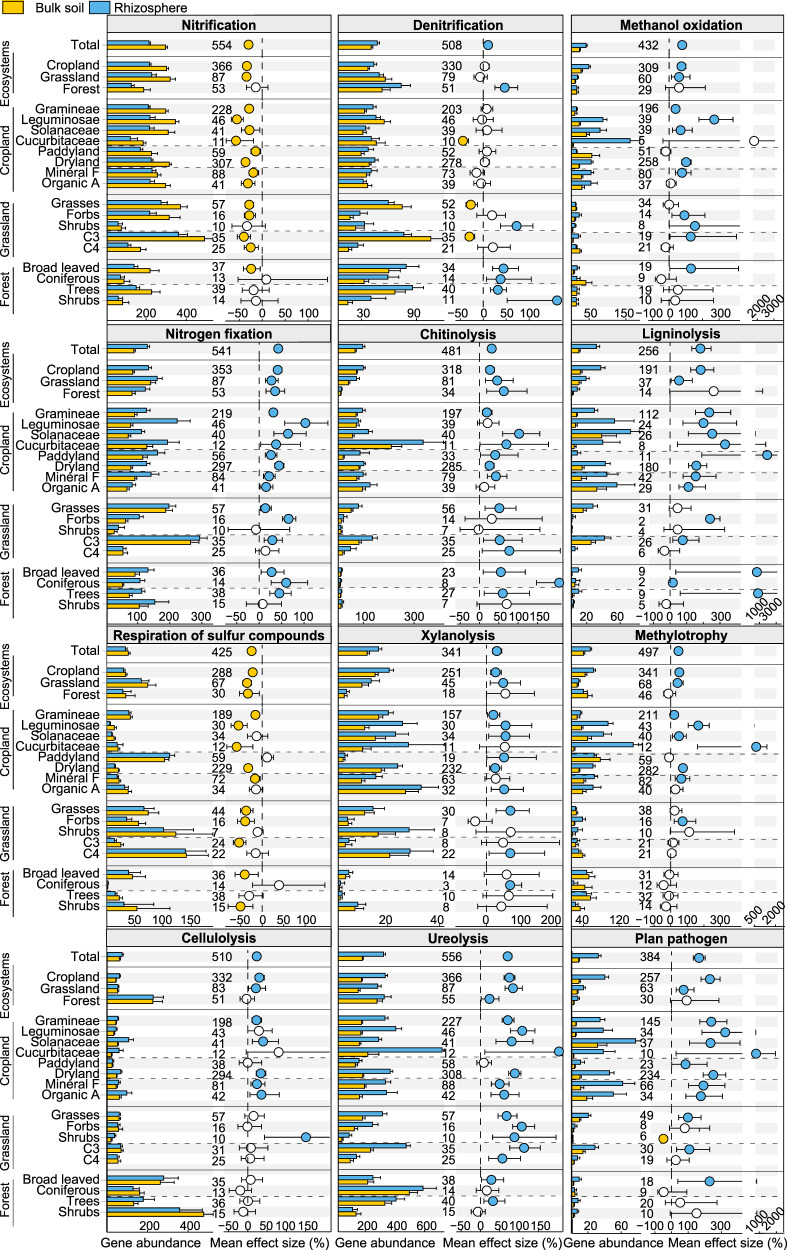
Functional potentials of bacterial communities in the rhizosphere and bulk soils. The Faprotax annotated function differences related to nitrification, denitrification, methanol oxidation, nitrogen fixation, chitinolysis, ligninolysis, respiration of sulfur compounds, xylanolysis, methylotrophy, cellulolysis, ureolysis, and plant pathogens. Bar charts reflect mean value and standard errors for each category. The error bars on the columns represent the standard errors (SE). All dots represent the percentage change of the effect size between bacterial community function potentials in the rhizosphere and bulk soils with 95% confidence intervals (CIs). Mean values <0 indicate a higher function in bulk soil (yellow dots: depletion in the rhizosphere), while mean values >0 indicate a higher function in the rhizosphere (blue dots). The intersection of the error bar with the zero line indicates that there is no significant difference of the function between bacterial communities in the rhizosphere and bulk soils (open dots). Sample size is showed by number of data pairs for each group. Mineral F and Organic A indicate the types of fertilization, which are mineral-only fertilization and organic amendments, respectively. Source data are provided as a Source data file.

All bacterial metabolic and ecologically relevant functions were predicted by the Functional Annotation of Prokaryotic Taxa (FAPROTAX). Low publication bias was also confirmed by the funnel plot with Egger’s regression test and the trim-and-fill analysis (Supplementary Fig. 10 and Supplementary Table 1). Compared to bulk soil, rhizosphere-inhabiting bacteria showed higher functional potentials of denitrification (+11%), cellulolysis (+23%), xylanolysis (+29%), chitinolysis (+32%), nitrogen fixation (+42%), methylotrophy (+47%), ureolysis (+70%), methanol oxidation (+78%), ligninolysis (+182%), and plant pathogenesis (+165%) (Fig. 5). In contrast, in the rhizosphere, the functional potential associated with nitrification was decreased by 29% (Fig. 5). The effects of the rhizosphere on functions associated with denitrification, methylotrophy, cellulolysis and ureolysis were absent in paddies. Remarkably, some plant groups in cropland and grassland ecosystems strongly altered the general effects on functions related to denitrification, which, however, were always significantly richer in the rhizosphere of forest plants. In forest ecosystem, the significant rhizosphere effects on some functions related to C cycling, including methanol oxidation, ligninolysis, xylanolysis, methylotrophy, and cellulolysis, were disappeared. Consequently, forest ecosystem showed a peculiar response where the rhizosphere had effect on these functions. The rhizosphere effects on chitinolysis, methylotrophy, and methanol oxidation could also be reduced by organic amendments. These results indicate that land use, plant groups, and fertilization regimes shape the differences between the rhizosphere and bulk soils.

## Discussion

### Bacterial diversity and composition in the rhizosphere and bulk soil

This meta-analysis is based on pairwise data (rhizosphere vs. bulk soils) from amplicon sequencing approaches to characterize taxonomic and functional features. The meta-analysis provided fundamental insights into the plant rhizosphere microbiome on an intercontinental scale, revealing plant-driven microbial taxa and their functional properties in this unique but cohesive habitat. The design of the meta-analysis allowed us to examine the general effects of plants on the rhizosphere bacterial communities across broad range of soil properties and geographic environments. This highlights the benefit of using sequencing data to synthesize general microbiome patterns and to indicate specialized functions and life strategies of microbial taxa based on niche differences between the rhizosphere and bulk soils.

The fact that rhizosphere microbiota differs from bulk soil microbiota is well documented^12,16,26–28^, and this is attributed to significant differences in physico-chemical properties driving niche differentiation^4,21,29,30^. In addition to environmental differences between niches, the general contrast between bulk soil and the rhizosphere was a very important cause of differences in microbiota composition^12,31^. Bacterial observed species richness, Shannon’s diversity index, and Faith’s phylogenetic diversity in rhizosphere were generally lower than in bulk soil under all environments. Thus, bacterial diversity decreases as substrate availability increases, a condition that is common in the rhizosphere. The general view is that the rhizosphere microbiota, a subset of the community in bulk soil, has certain similar traits in all plants. This underlines the selective effect of the rhizosphere, which has some general consequences on plant rhizobacterial assemblages. This holds true even for bacteria belonging to broad range of classes, orders and families. Although the magnitude of the effects differs between plants groups (Fig. 1), we emphasize that, even when genotypic and environmental differences were taken into account, certain similarities in the selection of microorganisms common to the rhizosphere were still observed^4,8,32^.

Of course, specific environmental conditions will substantially change the magnitude of the rhizosphere effect: the rice rhizosphere is a very good example. Most of the effects common to the rhizosphere of other plants were canceled out in paddy soils. Paddy soils are often anaerobic, and rice plants, therefore, have a well-developed aerenchyma. Due to the release of oxygen around the roots, the Eh value and oxygen content of the rice rhizosphere are much higher than in the bulk soil^33,34^ and vary widely. Thus, the rice rhizosphere is inhabited by a wider phylogeny of both anaerobic and aerobic bacteria. These results indicate that environmental heterogeneities, such as root exudates, Eh, and soil moisture changes, interact to give rise to selective effects in the rhizosphere.

Specific microbial taxa recruited to the rhizosphere from soil reservoirs can apparently form a distinct core microbiome^12,14^. The core microbiome around the roots contributes to plant growth, and fitness^17^. To date, the core microbiome of plants, whether in the rhizosphere, endosphere, or phyllosphere, has been defined primarily on the basis of taxonomic markers. However, we emphasize that more attention should be directed to identifying microbes with common functions that are selected for in the general rhizosphere environment. Thus, defining the microbiome based on function should make it easier to manipulate the community for useful purposes. The present comprehensive analysis revealed several predominant taxa that are consistently enriched in the rhizosphere, including the phyla Bacteroidetes and Proteobacteria (Fig. 2). This result underscores the fact that these phyla are generally adapted to C-rich conditions (common in the rhizosphere) for high metabolic activity, fast growth and propagation^30,35^, and are consequently very similar across diverse plant species. They are generally considered to be copiotrophs, or weedy fast-growing microbiota whose populations fluctuate opportunistically^28^. In contrast to the rhizosphere, bulk soils are generally enriched by other dominant phyla including Acidobacteria (Fig. 2b), which are oligotrophs^36,37^. Interestingly, several phylum-level taxa are similar between the rhizosphere and bulk soil, but differences are seen at finer taxonomic resolution (Fig. 2). Consequently, the general patterns that emerge based on the selective effect of the rhizosphere depend on taxonomic resolution and fundamental niches at the level of classes and families.

### Microbiome formation: from structure to functions

In microbial networks, highly interconnected species are grouped into modules, in which species interact more frequently and intensively than in the rest of the community. The modularity of the rhizosphere bacterial network is higher than that of the bulk soil (Fig. 3a, b). Since modules can be interpreted as microbial niches^38,39^, one possible explanation would be that niche differentiation is more pronounced in the rhizosphere^25^—both spatially and temporally. Modularity is one of the main organizing principles of biological networks^40^, and the higher modularity of the rhizosphere may indicate a more complex topological structure. Despite the higher modularity, the bacterial co-occurrence network in the rhizosphere is less stable (Fig. 3c), because the rhizosphere is characterized by very high temporal dynamics compared to the more static conditions in the bulk soil^30,41^. Plant species selectively enrich specific microorganisms by investing in root exudates to feed their rhizosphere microbiota^1,42^. The structure of the indigenous rhizosphere microbial community often varies considerably across host species^43^. In the soybean rhizosphere, the microbial community was selected through niche filtering, whereas the bulk soil community arose through neutral (stochastic) processes^44^. The rhizosphere network allocates more modules for executive functions, but fewer species for network robustness, partly reflecting the rapid element cycling in the rhizosphere^45,46^.

The rhizosphere and bulk soil are characterized by different microbial dormancy-dominating strategies: sporulation factors and toxin–antitoxin systems (Fig. 4a, b). Sporulation factors were more abundant in the bulk soil, while toxin–antitoxin systems were enriched in the rhizosphere (Fig. 4a, b). During plant growth, roots actively and passively release a wide range of organic compounds into the rhizosphere. These compounds are the driving force of microbial growth and activity^29,47,48^. The sporulation factor was abundant in the bulk dryland soils but was not significant in paddies (Fig. 4b). Hence, bacterial sporulation is more common in dryland soils because the environmental conditions in paddies are more stable and homogeneous, and paddies are always moist. The lower importance of sporulation in the rhizosphere (compared to bulk soil) indirectly confirmed the buffered amplitude of moisture variation associated with the conditions of mucilage swelling^49–51^. Genes related to dormancy/sporulation increase strongly with aridity^52^. Toxin–antitoxin systems consist of genes that encode a toxin protein that inhibits cell growth and an antitoxin that counteracts the toxin^53^.

It is generally assumed that rRNA operons in prokaryotic organisms are able to reflect their heterotrophic strategies^54^. Copiotrophs (*r*-strategists) have a high ribosome content, in part, by maintaining multiple ribosomal RNA operon copies in their genomes to achieve high growth rates^55^. The rhizosphere was inhabited by a greater number of copiotrophs (e.g., Bacteroidetes and Proteobacteria), as confirmed by higher rRNA operon counts associated with them (Fig. 4c). These results confirm that the major groups in the rhizosphere are fast-growing bacteria, especially the phyla Proteobacteria and Bacteroidetes^56^. Oligotrophs (K-strategists) have lower operon counts than copiotrophs^55^. A lower rRNA operon copy number is common in oligotrophic microbiota indicating slower growth rates and more stable populations. A functional feature of bacteria is that the copy number of the rRNA operon increases in response to the availability of resources^24^. Organisms with multiple operons are referred to *r*-strategists and tend to dominate in resource-rich environments and respond more rapidly to nutrient inputs^57–59^. Therefore, copiotrophs will predominate when resources are abundant, such as in rhizosphere habitats where plants secrete photosynthates to produce available C and energy.

As the interface between roots and soil, the rhizosphere hosts an abundant and diverse bacteriome that drives the soil C and N dynamics. Genes related to C and N transformation were all broadly higher in the rhizosphere (Fig. 5), due to the larger amount of plant-derived organic compounds, leading to higher microbial activity and abundance in the rhizosphere. This is directly confirmed by the fact that the rhizosphere is rich in almost all N-cycling functions (except nitrification), and the magnitude of their effects is greatly dependent on plant groups in agroecosystems (Fig. 5). Nitrification is prone to take place in aerobic conditions, but the rhizosphere generally suffers from oxygen deficiency^60^ because roots and microorganisms consume more oxygen than the bulk soil. Likewise, activities related to the decomposition and transformation of organic compounds, such as cellulolysis, xylanolysis, ligninolysis, ureolysis, and chitinolysis were generally more intense in the rhizosphere, reflecting the higher abundance and activity of bacteria degrading these substances. Methanol oxidation and methylotrophy genes are much more abundant in the rhizosphere than in the bulk soil (except in rice paddies). Methylotrophy is higher in the rhizosphere, but in paddy soils this difference is smoothed out because of the aerobic microenvironment around rice roots and the high dilution of available reduced C in water. Functional groups of plants, such as grasses and forbs, have distinct characteristics and fill specific niches^56,61^. Grasses have a much denser root systems, finer roots, and higher root biomass than forbs^62^, and thus accelerate nutrient cycling by intense litter and rhizodeposition decomposition. Hence, plant functional groups are likely to be critical drivers of rhizosphere functions.

Particularly in the rhizosphere, plants are continuously challenged by thousands of microbial populations, including commensals, pathogens, and symbionts. Plant pathogens and N-fixers (e.g., *Rhizobium* sp., etc.) are enriched in the rhizosphere (Fig. 5) because their reproduction and functioning depend on the supply of organic matter from the plant host. Although the rhizosphere is a dynamic environment, and the microbiome evolves rapidly in space and time, evidence is accumulating to confirm that plants shape the rhizosphere microbiome to their own benefit and skillfully utilize the microbial functional repertoire^13^. As expected, plant pathogens were more enriched in the rhizosphere than in the bulk soil, which may be due to the following reasons: (1) most plant pathogens grow saprophytically in the rhizosphere and derive their basal energy from the roots^63^; (2) bacterial pathogens, in particular, require a wound or natural opening to penetrate into the plant before they establish a parasitic relationship; (3) only a few groups of pathogenic bacteria are considered to be soilborne, probably because non-spore forming bacteria cannot survive well in bulk soils for long periods^64^; (4) certain pathogens, either candidate symbionts or stealthy pathogens, favor the colonization of the rhizosphere^65,66^. The rhizosphere is both the playground where soilborne pathogens infect plants and the battlefield where the complex rhizosphere community, including both microflora and microfauna, interacts with pathogens and influences the outcome of infection^64^. The number and diversity of deleterious and beneficial microorganisms are related to the quantity and quality of rhizodeposits and the outcome of microbial interactions in the rhizosphere. Nevertheless, identifying the equilibrium conditions for plant fitness remains a challenge, as does establishing a balance between passive attack by pathogens and active recruitment of beneficial bacteria.

**Fig. 6:**
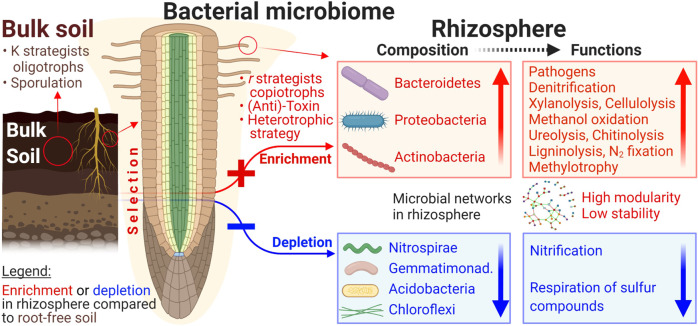
Conceptual figure showing the enrichment (red) and depletion (blue) of bacterial community taxa and functions in the rhizosphere relative to bulk soil. The vertical arrows correspond to the intensity of the changes. The light peach-colored area around the root reflects the enrichment with available organics caused by exudates.

By integrating sequencing data from multiple studies, we have generalized the main differences in the rhizosphere and bulk soil microbiomes with respect to bacterial diversity, composition, selection of specific groups, co-occurrence network, and a very broad range of functions (Fig. 6). The bacterial diversity in the rhizosphere is reduced by 0.9–5.3% and represents a subset of the bulk soil community. The bacterial community in the rhizosphere is highly enriched in copiotrophs such as Proteobacteria and Bacteroidetes, while bacteria such as Chloroflexi, Acidobacteria, and Nitrospirae are significantly reduced. Because of the surplus of organic C in the rhizosphere and rapid nutrient cycling, fast-growing bacteria have an excess of functions related to C transformation and plant pathogenesis, but the functions responsible for nitrification are depleted. Indirect evidence supporting the generalizations presented here is that land use regimes and plant functional groups influence almost all rhizosphere effects on bacterial diversity and functions. Based on our results, we validly sketched the generalized rhizosphere effects on bacteriome across continents, even though the soil properties and geographic distance showed certain contributions on the bacteriome variation (Supplementary Fig. 11). The selective influence of the rhizosphere on the formation of microbial communities overshadows to some extent the differences in soil, plant, or climate even at continent scale. This suggests that the rhizosphere is the powerful factor shaping the composition, structure, and functions of the soil microbiome and, thus, a key factor in the cycling of biogenic elements. Therefore, the present study expands our knowledge of the critical role of the rhizosphere effects in recruiting bacterial populations.

With the rapid development of instrumental and molecular techniques, there have been many attempts to consider the functional traits of microorganisms at the community level based on their ecological relevance. With further expansion of available soil metagenomic/metatranscriptomic datasets and the coupling of sequencing with isotopic probing approaches, more accurate and quantitative results are expected to be very promising in the future. Further efforts may still be needed to identify functions of plants and microbial communities influencing the rate of relevant soil processes at the ecosystem level, with a focus on plant performance and anthropogenic disturbances, to identify strategies to control or reshape the rhizosphere microbiome for microbial benefits to efficient nutrient cycling and soil health.

## Methods

### Data collection

An extensive literature survey was conducted through the Web of Science database (http://apps.webofknowledge.com/) until July 2021. The key words of the literature search were “bulk”, “rhizosphere”, and “bacteria”. A total of 122 publications were collected (see Supplementary information; Supplementary Fig. 12), comprising 557 pairs of bulk soils versus rhizosphere (see Source Data). In these studies, bacterial communities were investigated by high-throughput sequencing, including Illumina, Ion S5, and 454 platforms.

The following criteria were used to select suitable studies: (1) field or greenhouse experiments and studies with natural or planted vegetation were included; (2) studies for which sequencing metadata were not available from public repositories or upon request from individual study authors were excluded; (3) articles with a one-to-one correspondence of sequencing data between rhizosphere and bulk soil were included. Twenty-two primer pairs were identified from the study metadata (see Source data), and most samples used primer sets 515F and 806R^28^. In addition to sequencing data, we also collected the following parameters: plant species, ecosystem type (cropland, forest, and grassland), crop family (including Gramineae, Leguminosae, Solanaceae, Cucurbitaceae, and others), crop management (dryland, paddyland), fertilizer type in cropland (chemical fertilizer or organic fertilizer), group of herbaceous plants (grasses, forbs, and shrubs), grass photosynthetic pathway (C3, C4), forest classification (trees, shrubs), forest leaf traits (broad leaved, coniferous), location (i.e., latitude and longitude), mean annual temperature (MAT), mean annual precipitation (MAP), and soil properties (pH, available P, available K, SOC, total N, C:N, NH_4_^+^-N, and NO_3_^−^-N).

### Bioinformatics analysis and taxonomical annotation

We downloaded raw sequencing data from NCBI according to accession number, followed by FASTQ files from each study. The raw sequences were processed using the DADA2 pipeline^67^, which is designed to obtain sequences with a single-nucleotide difference, known as amplicon sequence variants (ASVs), rather than clustering similar sequences into operational taxonomic units (OTUs). Sequence reads were first filtered using DADA2’s recommended parameters. Potential primers were screened and removed. Filtered reads were then de-replicated and de-noised using DADA2 default parameters. Finally, paired-end sequences were merged and chimeras were removed. For each study, reads were truncated at the quality control score of 25. To integrate studies using different 16S rRNA gene regions, we adopted a closed-reference workflow, a database-dependent approach that uses a predefined set of reference sequences with a known taxonomy to obtain representative sequences and assign the taxonomy. Briefly, the USEARCH *closed_ref* command (sequence identity ≥ 97%) was used to map the above-obtained fragments to non-redundant full-length 16S rRNA sequences using the Silva 13.2 version database (http://www.drive5.com/usearch/manual/sintax_downloads.html). Fragments that could not be mapped were defined as unknown sequences. The ASV table was then generated according to the mapped full-length 16S sequences. The mapping ASV result of each data set in USEARCH table format was imported directly into the R software (version 3.6.0, R Core Team, 2019) and merged into the integrated ASV table for further analyses. Singleton ASVs and ASVs that were present only in one sample were removed. ASVs assigned to “Mitochondria”, “Chloroplast”, “Archaea”, and “Eukaryota” were removed from the bacterial community (proportion of every part showed in Supplementary Table 3). Finally, for downstream analysis, all samples were rarefied to the same sequencing depth (10,000 reads per sample) and alpha diversity comparisons between the rhizosphere and bulk soils were made using the following diversity measures: observed species richness, Shannon’s diversity index, Pielou’s evenness, and Faith’s phylogenetic diversity^68^. Bray-Curtis dissimilarity matrix and principal coordinates analysis (PCoA) were used to estimate and visualize beta diversity of samples. Statistical analysis of beta diversity was performed by permutational multivariate analysis of variance (PERMANOVA).

### Network construction and analysis

Microbial co-occurrence networks were constructed using Spearman’s correlation method to identify pairwise associations of ASVs^69^. ASVs with relative abundances < 0.02% were removed. *P* values were corrected for multiple comparisons using the Benjamini–Hochberg false discovery rate (FDR) control procedure^70^. Spearman correlation thresholds were determined using the Random matrix theory (RMT) method^71^. Finally, only correlations with an adjusted *p* value below 0.05 and a score above the threshold were retained. Network properties were calculated using the igraph package of the R software. Network visualization was performed using Cytoscape 3.7.2^72^. The datasets were divided into different plant groups to established co-occurrence networks and visualized in Gephi 0.9.2^73^. To compare the stability of the networks, we used natural connectivity, a new structural robustness metric that can sensitively and reliably assess the structural robustness of networks. It is an average eigenvalue derived from the network spectrum, which describes the redundancy of alternative paths^74^. The stability of each bacterial network was assessed by removing nodes from the static network to evaluate how quickly the robustness degraded. Briefly, all nodes were sorted by betweenness from small to large, and then nodes were removed sequentially until 80% of the nodes were removed^75^. Network modularity for each network was characterized. A module is a group of nodes (i.e., ASVs) that are highly connected within the group and less connected outside the group^76^. Modules were detected using the greedy modularity optimization method^77^. The connectivity of each node was determined based on its within-module connectivity (Zi) and among-module connectivity (Pi)^78^, which were then used to classify its topological role in the network. All nodes were categorized into four subcategories: module hubs (nodes that are highly connected within a module, Zi > 2.5 and Pi < 0.62), network hubs (nodes that are highly connected within or among modules, Zi > 2.5 and Pi > 0.62), peripherals (nodes with few connections within or among modules, Zi < 2.5 and Pi < 0.62) and connectors (nodes that are highly connected among modules, Zi < 2.5 and Pi > 0.62)^79,80^.

### Prediction of functions and analysis of dormancy potential and heterotrophic strategy

The Functional Annotation of Prokaryotic Taxa v.1.0 (FAPROTAX, http://mem.rcees.ac.cn:8080/) pipeline was used to extrapolate bacterial community functions. FAPROTAX was constructed by integrating multiple culturable prokaryotic bacteria with reported functions and contained more than 7600 functional annotations for more than 4600 species. This makes it a powerful tool to perform functional annotation based on published metabolic and ecological functions such as nutrient (e.g., C, N, P, and S) cycling, plant pathogens, and symbionts^81^. The dormancy potential and heterotrophic strategy were determined using the PICRUSt2 with the Integrated Microbial Genomes (IMG) database^82^. Approximately 0.35% of the ASVs were above the Nearest Sequenced Taxon Index (NSTI) cut-off score of values >2, and these ASVs were removed. Dormancy potential was measured as the abundances of genes (including genes involved in toxin–antitoxin systems and sporulation factors) that confer dormancy strategies^54^. This reflects the ability of microorganisms to decrease metabolic activity and persist in adverse conditions^83^. The weighted mean ribosomal operon copy number (operon count) was calculated as a proxy for microbial heterotrophic strategy^57^, which reflects the rapid response of microbes to resources^54^. Copiotrophs are assumed to have relatively higher operon counts than oligotrophs^55^.

### Calculation of the response ratio

The natural log-transformed response ratio (lnRR) was employed as a metric to estimate the rhizosphere effect sizes on bacterial community characteristics (including observed species richness, Shannon’s diversity index, Pielou’s evenness, Faith’s phylogenetic diversity, dormancy potential, heterotrophic strategy, and Faprotax predicted functions).

1 $${{{{{\rm{The}}}}}}\,{{{{{\rm{lnRR}}}}}}\,{{{{{\rm{was}}}}}}\,{{{{{\rm{calculated}}}}}}\,{{{{{\rm{as}}}}}}:{{{{{\rm{lnRR}}}}}}=\,{{{{{\mathrm{ln}}}}}}({X}_{t}/{X}_{c})$$

where ${X}_{t}\,$ and ${X}_{c}$ stand for mean values of a given variable of the rhizosphere and bulk soil groups, respectively^84^. The variances ($v$) of each lnRR were calculated as:

2 $$v=\frac{{S}_{t}^{2}}{{n}_{t}{X}_{t}^{2}}+\frac{{S}_{c}^{2}}{{n}_{c}{X}_{c}^{2}}$$

Where *n*_*t*_ and *n*_*c*_ are the sample sizes, and *S*_*t*_ and *S*_*c*_ are the standard deviations of the means in the rhizosphere and bulk soil groups, respectively. To determine whether the rhizosphere and bulk soil compartments had a significant effect on the variable, we employed a random-effects model using Metawin (Version 2.1.4)^85^. Our analysis included two sources of variance, including within-study variance (ν) and between-study variance (τ^2^), both of which were used as a weighting factor [w = 1/ (v + τ^2^)] to calculate mean lnRR values. Bootstrapping with 999 iterations was performed to estimate the 95% confidence intervals (CIs). If the 95% CI values for the effect size of a variable did not overlap with zero, the effect size of the variable was considered to be significantly different between the rhizosphere and bulk soil. For a better understanding, the weighted mean effect size was converted back into a percentage change using the formula:

3 $$\left({e}^{\overline{{ln}R{R}^{{\prime} }}}-1\right)\times 100 \%$$

To avoid unreliable results, some subgroups with an insufficient sample size (*n*  <  10) were excluded from the categorical analysis. In the categorical group analysis, the total heterogeneity among studies (Q_T_) was partitioned into within-group heterogeneity (Q_W_) and between-group heterogeneity (Q_B_). A significant Q_B_ means a significant difference in the mean effect size between different groups in each category (Supplementary Tables 4–7). The publication bias was tested with the funnel plot using the “metafor” package^86^. Possible publication bias was statistically tested using Egger’s regression test^87^. If there were indications of publication bias, the trim-and-fill method was applied to estimate the number of potentially missing studies, and then fill the missing data in, to examine whether any results changed substantially. Model selection was performed to identify the most important predictors for natural log-transformed response ratios of bacterial diversity (i.e., observed species richness, Shannon’s diversity index, Pielou’s evenness and Faith’s phylogenetic diversity) between bulk soil and rhizosphere using the “glmulti” package in R^88^. The importance value of each particular predictor was expressed as the sum of the Akaike weights for models included this factor, which can be considered as the overall support for each predictor across all models. A cutoff of 0.8 was set to differentiate between important and unimportant predictor. Six types of candidate predictors were considered in the model selection analysis, that are, location (longitude and latitude), ecosystems, soil properties (pH, SOC, and total N) (Supplementary Fig. 13). Since only a few studies simultaneously measured available P, available K, NH_4_^+^-N and NO_3_^−^-N in both bulk and rhizosphere soils, these properties were not considered in the model selection.

### Taxa with significant differences between the rhizosphere and bulk soil

The ALDEx2 tool was used to determine differences at phylum and ASV levels in terms of relative abundance between certain pairwise comparisons (rhizosphere versus bulk soil)^89^. The counts for taxa that were retained after filtration were tested using the default parameters of the aldex function (including mc.samples = 128, test = “t”, denom = “all”). The aldex function takes the counts of reads as input and performs a centered log-ratio (CLR) transformation to infer abundance prior to performing statistical testing. Wilcoxon’s rank-sum tests were used, followed by a Benjamini–Hochberg false discovery rate (FDR) correction to identify taxa with significantly different relative abundances between bulk and rhizosphere soils, using the ALDEx2 “aldex.ttest” function. Treemap (Version 4.1.1) software was used to visualize the significantly different ASVs as bubble diagrams, where the size of the bubble indicates the size of the ALDEx2 effect. In addition, a phylogenetic tree was generated using the MUSCLE software^90^ and phylogenetic groups were delimited in the resulting tree using iTOL^91^.

### Reporting summary

Further information on research design is available in the Nature Research Reporting Summary linked to this article.

## Supplementary information

Supplementary information

Reporting Summary

## Data Availability

The sequences used in this study are previously published and publicly available sequences that were downloaded from the NCBI Sequence Read Archive (https://www.ncbi.nlm.nih.gov/). All accession numbers are listed in Source data. Source data are provided with this paper.

## Source data

Source data

## Code availability

The scripts for analysis are available on https://zenodo.org/badge/latestdoi/430344587.
