# Supplementary material for: Rhizosphere bacteriome structure and functions
Source: Nat Commun. 2022 Feb 11;13:836. doi: 10.1038/s41467-022-28448-9 (PMC8837802; doi:10.1038/s41467-022-28448-9)
Supplement: Supplementary file 1 — Supplementary information [file 41467_2022_28448_MOESM1_ESM.pdf]

Supplementary Information

Bacterial Microbiome of the Rhizosphere: from Structure to Functions

Ning Ling [a, b, *], Tingting Wang [b], Yakov Kuzyakov [c, d, **]

a, Centre for Grassland Microbiome, State Key Laboratory of Grassland Agro-ecosystems, College of Pastoral Agricultural Science and Technology, Lanzhou University, Lanzhou, 730020, Gansu, China

b, Jiangsu Provincial Key Lab for Organic Solid Waste Utilization, Jiangsu Collaborative Innovation Center for Solid Organic Waste Resource Utilization, Nanjing Agricultural University, Nanjing, 210095, China

c, Department of Soil Science of Temperate Ecosystems, Department of Agricultural Soil Science, University of Goettingen, 37077, Göttingen, Germany

d, Peoples Friendship University of Russia (RUDN University), 117198 Moscow, Russia

Corresponding authors:

* Ning Ling, E-mail address: nling@njau.edu.cn

** Yakov Kuzyakov, E-mail address: kuzyakov@gwdg.de

Supplementary Tables:

Supplementary Table 1. Summary table of response ratios from the original meta-analysis as well as from trim and fill test.

Supplementary Table 2. Major properties of bacterial networks in bulk soil and the rhizosphere.

Supplementary Table 3. The proportions of removed sequences and unknow sequences during the processing of sequencing data.

Supplementary Table 4. Heterogeneity statistics of categorical group analyses on bacterial alpha diversity.

Supplementary Table 5. Heterogeneity statistics of categorical group analyses on bacterial alpha diversity depending on experiment management, sequencing platform and target regions of primer pairs.

Supplementary Table 6. Heterogeneity statistics of categorical group analyses on dormancy potential and heterotrophic strategy.

Supplementary Table 7. Heterogeneity statistics of categorical group analyses on bacterial community function.

Supplementary Figures:

Supplementary Figure 1. Map of sampling sites from 122 independent studies with 557 pairs from rhizosphere and bulk soils for data merging.

Supplementary Figure 2. Funnel plots for bacterial diversity between bulk soils and rhizosphere.

Supplementary Figure 3. Diversity of bacterial communities between bulk soils and rhizosphere under different experiment management, sequencing platform and target regions.

Supplementary Figure 4. Bacterial community composition of bulk soils and rhizosphere.

Supplementary Figure 5. The phyla differences between bacterial communities in rhizosphere and bulk soils depending on plant groups.

Supplementary Figure 6. Phylogeny of microorganisms with significant differences in bulk soils and rhizosphere bacterial communities.

Supplementary Figure 7. Phylogeny of microorganisms with significant differences in bulk soils and rhizosphere bacterial communities depending on plant groups.

Supplementary Figure 8. Co-occurrence networks of bacterial ASVs in bulk soils and rhizosphere depending on plant groups.

Supplementary Figure 9. Funnel plots for dormancy potentials and heterotrophic strategies of bacterial communities in rhizosphere and bulk soils.

Supplementary Figure 10. Funnel plots for the functional potentials of bacterial communities in rhizosphere and bulk soils.

Supplementary Figure 11. Relationship between microbial community similarity and geographical distance as well as the contributions of different factors to bacterial community variation.

Supplementary Figure 12. PRISMA flow diagram for the studies selected and included in the systematic review.

Supplementary Figure 13. Model-averaged importance of the predictors for natural log-transformed response ratios of bacterial diversity between bulk soils and rhizosphere.

List of publications used in this synthesis

Supplementary Table 1. Summary table of response ratios from the original meta-analysis as well as from trim and fill test. Response ratio with 95% CIs were represented.

	Original Meta analysis			Trim and fill test		
	RR	CI Low	CI High	RR	CI Low	CI High
Shannon's diversity index	-0.0092	-0.0148	-0.0037	-0.0079	-0.0135	-0.0022
Pielou's evenness	-0.0005	-0.0035	0.0025	0.0002	-0.0028	0.0032
Heterotrophic strategy	0.0638	0.0522	0.0754	0.0416	0.0265	0.0567
Nitrification	-0.3546	-0.4001	-0.3092	-0.3257	-0.3724	-0.2790
Denitrification	0.1292	0.0657	0.1927	0.1195	0.0556	0.1834
Chitinolysis	0.2729	0.2033	0.3426	0.2603	0.1905	0.3302
Respiration of sulfur compounds	-0.2816	-0.3409	-0.2223	-0.2390	-0.2997	-0.1782
Cellulolysis	0.2078	0.1410	0.2745	0.1770	0.1090	0.2449
Ureolysis	0.5318	0.4661	0.5974	0.5346	0.4690	0.6003

Supplementary Table 2. Major properties of bacterial networks in bulk soil and the rhizosphere.

		edges	nodes	average. Degree	modularity
Global	Bulk	3608	559	12.91	0.52
	Rhizosphere	2210	534	8.28	0.60
Gramineae	Bulk	3240	526	12.32	0.56
	Rhizosphere	2284	482	9.48	0.58
Leguminosae	Bulk	3111	492	12.65	0.70
	Rhizosphere	2579	475	10.86	0.74
Solanaceae	Bulk	3970	486	16.34	0.59
	Rhizosphere	4070	507	16.06	0.57
Cucurbitaceae	Bulk	2298	339	13.56	0.71
	Rhizosphere	1621	354	9.16	0.71
Grass	Bulk	4822	468	20.61	0.70
	Rhizosphere	4271	473	18.06	0.72
Forbs	Bulk	3088	404	15.29	0.60
	Rhizosphere	3797	437	17.38	0.63
Trees	Bulk	2857	473	12.08	0.60
	Rhizosphere	4018	491	16.37	0.63

Supplementary Table 3. The proportions of removed sequences and unknow sequences during the processing of sequencing data.

	Sequences proportion (%)
Singleton	0.0001
ASVs presented only in one sample	0.20
Archaea	0.88
Eukaryota	0.43
Chloroplast	0.90
Mitochondria	0.45
Unknown	14

Supplementary Table 4. Heterogeneity statistics of categorical group analyses on bacterial alpha diversity. Total heterogeneity (Q_T) was partitioned into within-group heterogeneity (Q_W) and between-group heterogeneity (Q_B). A significance of Q_B indicates that the effect sizes are significantly different between the levels of the categorical group. A two-sided *p* value <0.05 was considered to be statistically significant.

Response variables	Categorical variables	Q_T	Q_B	Q_W	*p* value (Q_B)
Observed species richness	Ecosystem type	533.19	15.47	517.71	0.001
	Crop family	379.67	17.88	361.79	0.003
	Crop management	449.20	3.35	445.85	0.083
	Fertilizer type in cropland	149.42	2.54	146.88	0.138
	Group of herbaceous plants	88.82	1.24	87.58	0.817
	Grass photosynthetic pathway	62.56	8.03	54.53	0.009
	Forest leaf traits	59.22	2.25	56.97	0.166
	Forest classification	62.90	5.41	57.48	0.063
Shannon's diversity index	Ecosystem type	986.88	55.65	931.23	0.001
	Crop family	698.67	68.79	629.89	0.001
	Crop management	758.99	5.85	753.14	0.094
	Fertilizer type in cropland	170.86	4.95	165.92	0.056
	Group of herbaceous plants	93.86	6.75	87.11	0.065
	Grass photosynthetic pathway	55.76	4.44	51.32	0.027
	Forest leaf traits	60.41	0.52	59.89	0. 502
	Forest classification	62.86	3.22	59.64	0.101
Pielou's evenness	Ecosystem type	551.10	59.58	1381.36	0.001
	Crop family	1194.48	141.3	1053.16	0.001
	Crop management	1267.26	1.76	1265.50	0.422
	Fertilizer type in cropland	368.17	12.58	355.59	0.053
	Group of herbaceous plants	187.12	19.43	167.69	0.007
	Grass photosynthetic pathway	189.28	5.94	183.34	0.082
	Forest leaf traits	30.20	0.27	29.94	0.540
	Forest classification	33.64	0.46	33.18	0.398
Faith's phylogenetic diversity	Ecosystem type	610.56	13.30	597.26	0.010
	Crop family	446.93	18.32	428.61	0.016
	Crop management	478.99	2.65	476.35	0.155
	Fertilizer type in cropland	141.22	3.41	137.81	0.079
	Group of herbaceous plants	52.47	2.48	49.99	0.186
	Grass photosynthetic pathway	55.24	2.32	52.92	0.144
	Forest leaf traits	67.11	7.29	59.82	0.055
	Forest classification	66.32	1.35	64.98	0.289

Ecosystem type: cropland, forest and grassland; Crop family: Gramineae, Leguminosae, Solanaceae, Cucurbitaceae; Crop management: dryland, paddyland; Fertilizer type in cropland: chemical fertilizer and organic fertilizer; Group of herbaceous plants: grasses, forbs and shrubs; Grass photosynthetic pathway:C3, C4; Forest classification: trees, shrubs; Forest leaf traits: broad leaved, coniferous.

Supplementary Table 5. **Heterogeneity statistics of categorical group analyses on bacterial alpha diversity depending on experiment management, sequencing platform and target regions of primer pairs.** Total heterogeneity (Q_T) was partitioned into within-group heterogeneity (Q_W) and between-group heterogeneity (Q_B). A significance of Q_B indicates that the effect sizes are significantly different between the levels of the categorical group. A two-sided *p* value <0.05 was considered to be statistically significant.

Response variables		Categorical variables	Q_T	Q_B	Q_W	*p* value (Q_B)
Observed species richness	Total	Sequencing platform	573.93	26.06	547.87	0.001
		Target regions	476.51	6.47	470.04	0.053
		Experimental management	583.67	2.91	580.76	0.121
	Cropland	Target regions	342.10	1.41	340.69	0.259
	Grassland	Target regions	84.02	11.18	72.84	0.003
	Forest	Target regions	56.61	0.17	56.44	0.722
Shannon's diversity index	Total	Sequencing platform	964.54	57.98	906.56	0.001
		Target regions	817.25	27.40	789.85	0.001
		Experimental management	604.79	13.26	591.54	0.398
	Cropland	Target regions	604.79	13.26	591.54	0.077
	Grassland	Target regions	82.40	0.13	82.27	0.002
	Forest	Target regions	54.89	2.08	52.81	0.183
Pielou's evenness	Total	Sequencing platform	1340.7	53.75	1286.95	0.060
		Target regions	1121.1	41.33	1079.80	0.001
		Experimental management	1408.7	0.12	1408.58	0.814
	Cropland	Target regions	988.51	30.78	957.73	0.001
	Grassland	Target regions	230.20	0.41	229.79	0.659
	Forest	Target regions	39.98	4.26	35.72	0.008
Faith's phylogenetic diversity	Total	Sequencing platform	633.77	38.33	595.44	0.001
		Target regions	541.27	2.57	538.70	0.152
		Experimental management	647.69	2.78	644.90	0.132
	Cropland	Target regions	418.55	0.57	417.98	0.502
	Grassland	Target regions	71.37	2.65	68.72	0.112
	Forest	Target regions	59.82	0.12	59.70	0.767

Ecosystem type: cropland, forest and grassland; Sequencing platform: Illumina and Ion S5; Target regions: V3~V4 and V4~V5; Experiment management: field and greenhouse.

Supplementary Table 6. Heterogeneity statistics of categorical group analyses on dormancy potential and heterotrophic strategy. Total heterogeneity (Q_T) was partitioned into within-group heterogeneity (Q_W) and between-group heterogeneity (Q_B). A significance of Q_B indicates that the effect sizes are significantly different between the levels of the categorical group. A two-sided *p* value <0.05 was considered to be statistically significant.

Response variables	Categorical variables	Q_T	Q_B	Q_W	*p* value (Q_B)
Toxin-Antitoxin	Ecosystem type	789.32	0.93	788.38	0.748
	Crop family	721.74	628.5	93.15	0.001
	Crop management	595.82	63.05	532.77	0.001
	Fertilizer type in cropland	178.91	0.60	178.31	0.519
	Group of herbaceous plants	95.34	5.09	90.25	0.102
	Grass photosynthetic pathway	111.96	24.02	87.94	0.001
	Forest leaf traits	92.99	4.46	88.53	0.135
	Forest classification	97.14	0.56	96.58	0.552
Sporulation	Ecosystem type	755.56	3.21	752.36	0.335
	Crop family	582.76	39.69	543.07	0.001
	Crop management	574.89	5.52	569.37	0.062
	Fertilizer type in cropland	171.97	3.30	168.66	0.103
	Group of herbaceous plants	110.76	8.85	101.91	0.061
	Grass photosynthetic pathway	64.98	0.02	64.98	0.958
	Forest leaf traits	77.53	2.52	75.01	0.184
	Forest classification	79.82	0.026	79.80	0.879
Heterotrophic-strategy	Ecosystem type	335.0	3.51	331.50	0.038
	Crop family	204.7	4.02	200.78	0.137
	Crop management	243.1	3.96	239.23	0.007
	Fertilizer type in cropland	322.8	8.78	314.02	0.059
	Group of herbaceous plants	156.1	7.30	148.83	0.040
	Grass photosynthetic pathway	120.5	8.73	111.77	0.015
	Forest leaf traits	66.98	0.00	66.98	0.952
	Forest classification	70.79	0.02	70.77	0.900

Ecosystem type: cropland, forest and grassland; Crop family: Gramineae, Leguminosae, Solanaceae, Cucurbitaceae; Crop management: dryland, paddyland; Fertilizer type in cropland: chemical fertilizer and organic fertilizer; Group of herbaceous plants: grasses, forbs and shrubs; Grass photosynthetic pathway:C3, C4; Forest classification: trees, shrubs; Forest leaf traits: broad leaved, coniferous.

Supplementary Table 7. Heterogeneity statistics of categorical group analyses on bacterial community function. Total heterogeneity (Q_T) was partitioned into within-group heterogeneity (Q_W) and between-group heterogeneity (Q_B). A significance of Q_B indicates that the effect sizes are significantly different between the levels of the categorical group. A two-sided p value <0.05 was considered to be statistically significant.

Response variables	Categorical variables	Q_T	Q_B	Q_W	p value (Q_B)
Nitrification	Ecosystem type	793.90	7.29	786.61	0.078
	Crop family	594.28	41.02	553.26	0.001
	Crop management	619.33	13.54	605.79	0.007
	Fertilizer type in cropland	185.29	2.46	182.83	0.206
	Group of herbaceous plants	88.64	0.13	88.50	0.924
	Grass photosynthetic pathway	71.28	1.59	69.69	0.217
	Forest leaf traits	75.86	1.58	74.28	0.239
	Forest classification	64.50	0.03	64.47	0.858
Denitrification	Ecosystem type	644.64	11.31	633.33	0.010
	Crop family	425.25	3.26	421.99	0.049
	Crop management	470.37	1.10	469.27	0.353
	Fertilizer type in cropland	178.03	0.47	177.56	0.531
	Group of herbaceous plants	116.34	13.19	103.15	0.004
	Grass photosynthetic pathway	77.07	6.22	70.85	0.024
	Forest leaf traits	65.48	0.30	65.18	0.577
	Forest classification	71.04	4.44	66.60	0.039
Methanol oxidation	Ecosystem type	670.32	0.40	669.92	0.888
	Crop family	586.63	54.13	532.50	0.001
	Crop management	559.41	36.89	522.52	0.001
	Fertilizer type in cropland	202.48	6.98	195.50	0.031
	Group of herbaceous plants	69.54	5.66	63.88	0.068
	Grass photosynthetic pathway	57.47	6.52	50.95	0.024
	Forest leaf traits	22.69	3.10	19.59	0.076
	Forest classification	25.96	0.03	25.93	0.884
Nitrogen fixation	Ecosystem type	717.65	2.66	714.99	0.335
	Crop family	512.82	34.86	477.96	0.001
	Crop management	527.67	2.94	524.73	0.162
	Fertilizer type in cropland	178.19	0.47	177.72	0.531
	Group of herbaceous plants	130.15	15.69	114.46	0.002
	Grass photosynthetic pathway	62.39	0.74	61.65	0.412
	Forest leaf traits	71.28	1.63	69.65	0.209
	Forest classification	77.27	2.75	74.52	0.110
Chitinolysis	Ecosystem type	503.30	3.25	500.05	0.488
	Crop family	320.91	20.76	300.15	0.001
	Crop management	347.66	0.56	347.10	0.466
	Fertilizer type in cropland	104.52	2.11	102.41	0.131

	Group of herbaceous plants	100.16	1.15	99.01	0.606
	Grass photosynthetic pathway	71.00	0.24	70.76	0.664
	Forest leaf traits	36.29	5.57	30.72	0.03
	Forest classification	35.36	0.03	35.33	0.89
Ligninolysis	Ecosystem type	416.44	7.48	408.96	0.110
	Crop family	262.24	0.57	261.67	0.952
	Crop management	370.18	26.05	344.13	0.001
	Fertilizer type in cropland	89.00	0.61	88.39	0.496
	Group of herbaceous plants	34.80	1.06	33.74	0.611
	Grass photosynthetic pathway	30.83	2.09	28.74	0.152
	Forest leaf traits	8.98	0.68	8.30	0.411
	Forest classification	13.12	2.83	10.29	0.126
Respiration of sulfur compounds	Ecosystem type	586.07	5.18	580.89	0.161
	Crop family	434.56	34.29	400.27	0.001
	Crop management	453.29	38.29	415.00	0.001
	Fertilizer type in cropland	131.04	0.05	130.99	0.803
	Group of herbaceous plants	81.81	78.96	2.85	0.594
	Grass photosynthetic pathway	58.66	8.60	50.06	0.013
	Forest leaf traits	31.82	2.05	29.78	0.182
	Forest classification	33.98	0.52	33.46	0.506
Xylanolysis	Ecosystem type	344.04	1.50	342.54	0.504
	Crop family	285.25	4.12	281.13	0.367
	Crop management	287.39	0.52	286.87	0.460
	Fertilizer type in cropland	115.36	114.6	0.80	0.415
	Group of herbaceous plants	43.90	6.01	37.89	0.086
	Grass photosynthetic pathway	22.57	0.07	22.50	0.790
	Forest leaf traits	16.80	0.01	16.79	0.958
	Forest classification	17.49	0.09	17.40	0.770
Methylotrophy	Ecosystem type	608.29	12.24	596.05	0.016
	Crop family	614.66	103.45	511.21	0.001
	Crop management	513.85	32.20	481.65	0.001
	Fertilizer type in cropland	145.04	1.53	143.51	0.287
	Group of herbaceous plants	66.73	3.18	63.5	0.217
	Grass photosynthetic pathway	41.61	0.13	41.48	0.702
	Forest leaf traits	52.64	1.05	51.59	0.383
	Forest classification	54.32	0.29	54.03	0.630
Cellulolysis	Ecosystem type	517.17	6.11	511.06	0.063
	Crop family	401.63	4.26	397.38	0.323
	Crop management	379.47	3.91	375.56	0.070
	Fertilizer type in cropland	115.97	0.37	115.60	0.539
	Group of herbaceous plants	106.31	9.14	97.17	0.032
	Grass photosynthetic pathway	67.29	0.01	67.28	0.967
	Forest leaf traits	38.47	1.12	37.35	0.270
	Forest classification	41.47	0.27	41.20	0.547

Ureolysis	Ecosystem type	433.66	9.21	424.45	0.005
	Crop family	292.23	10.27	281.96	0.026
	Crop management	283.26	16.93	266.33	0.001
	Fertilizer type in cropland	94.66	0.22	94.44	0.597
	Group of herbaceous plants	91.02	2.17	88.85	0.347
	Grass photosynthetic pathway	68.61	3.76	64.85	0.063
	Forest leaf traits	108.04	0.42	107.62	0.639
	Forest classification	115.13	4.53	110.60	0.103
Plant pathogen	Ecosystem type	466.16	14.14	452.02	0.004
	Crop family	300.24	12.49	287.75	0.621
	Crop management	343.16	5.31	337.85	0.050
	Fertilizer type in cropland	151.67	0.04	151.63	0.858
	Group of herbaceous plants	67.40	6.99	60.41	0.048
	Grass photosynthetic pathway	47.92	1.71	46.22	0.234
	Forest leaf traits	35.39	6.17	29.22	0.069
	Forest classification	32.67	0.47	32.20	0.537

Ecosystem type: cropland, forest and grassland; Crop family: Gramineae, Leguminosae, Solanaceae, Cucurbitaceae; Crop management: dryland, paddyland; Fertilizer type in cropland: chemical fertilizer and organic fertilizer; Group of herbaceous plants: grasses, forbs and shrubs; Grass photosynthetic pathway: C3, C4; Forest classification: trees, shrubs; Forest leaf traits: broad leaved, coniferous.

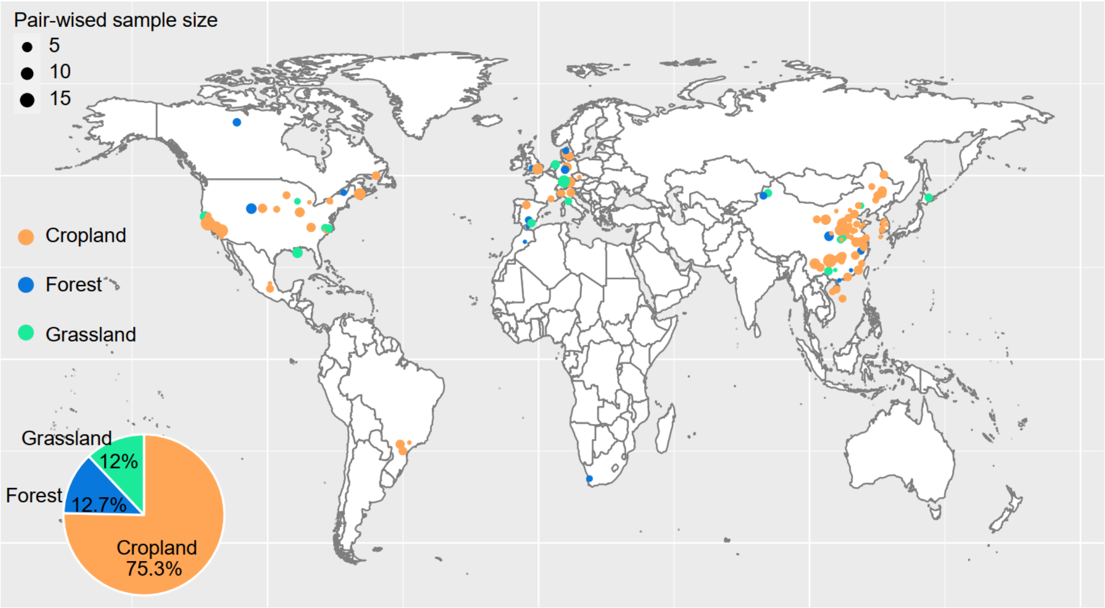

Supplementary Figure 1. Map of sampling sites from 122 independent studies with 557 pairs from rhizosphere and bulk soils for data merging. The locations of sampling sites are marked by circles on map. Circle colors represented different ecosystems. Orange, green, and blue in the pie diagram represent cropland, grassland and forest, respectively. Circle size represents the pair-wise sample size of the bulk soils and rhizosphere.

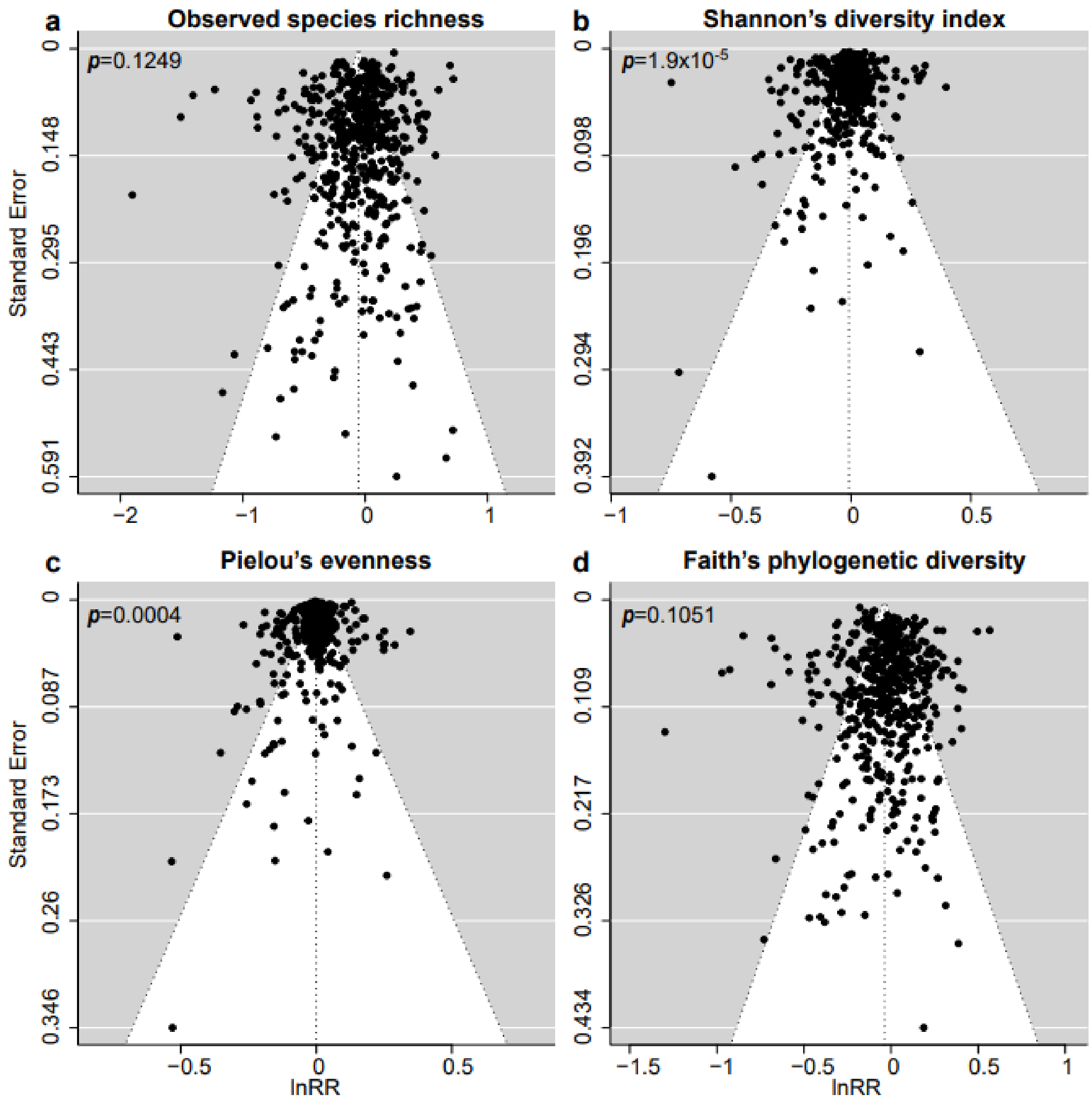

Supplementary Figure 2. Funnel plots for bacterial diversity between bulk soils and rhizosphere. A, Funnel plot of observed species richness; **b**, Funnel plot of Shannon's diversity index; **c**, Funnel plot of Pielou's evenness; **d**, Funnel plot of Faith's phylogenetic diversity. The top of each panel contains the results of publication bias tests using Egger's regression. A two-sided *p* value indicating statistical significance of the funnel plot asymmetry test. Asymmetry test showed a symmetric distribution ($p > 0.05$) in observed species richness (a) and Faith's phylogenetic diversity (d). Asymmetry test showed an asymmetric distribution ($p < 0.05$) in Shannon's diversity index (b) and Pielou's evenness (c). lnRR: natural log-transformed response ratio.

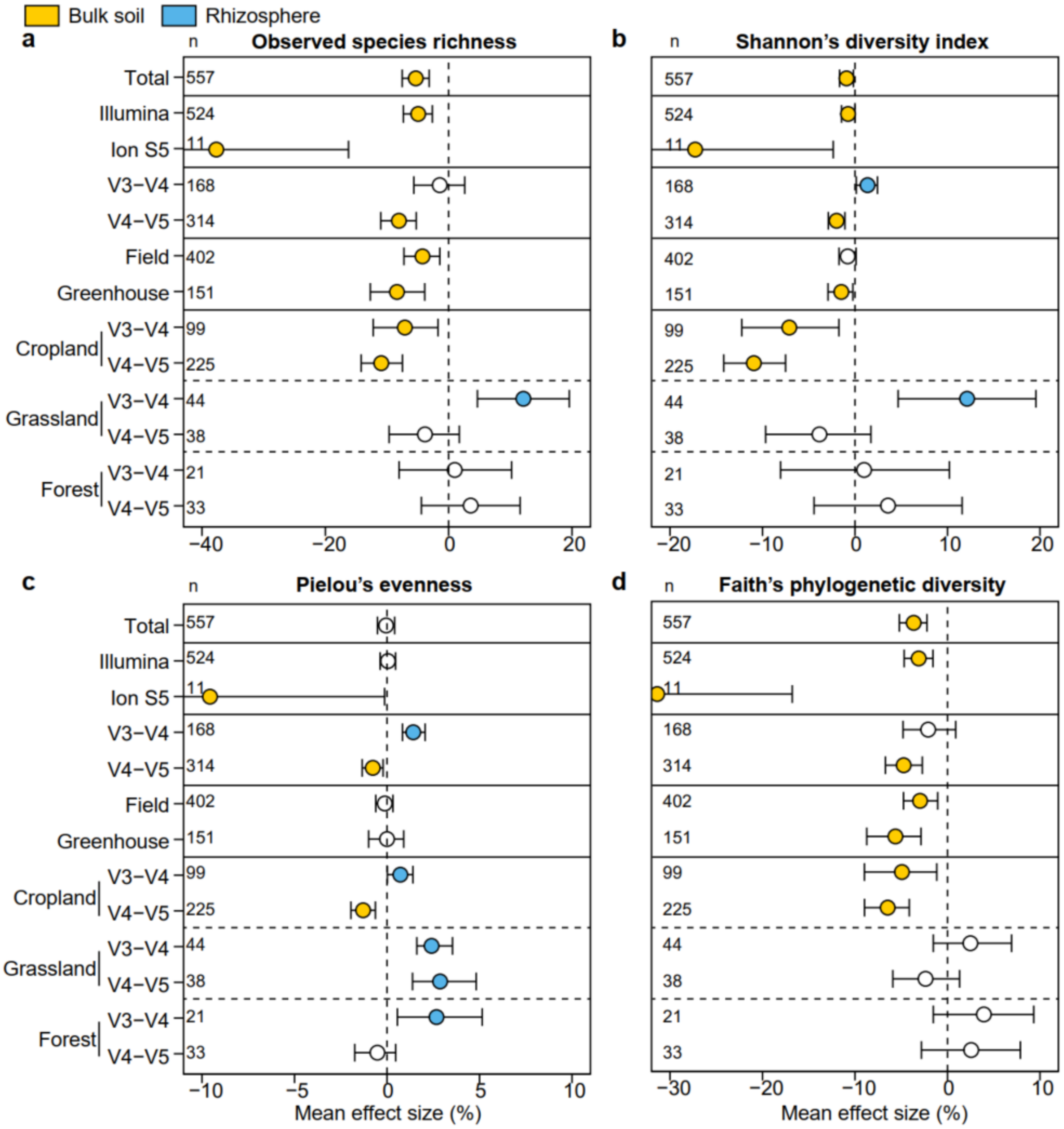

Supplementary Figure 3. Diversity of bacterial communities between bulk soils and rhizosphere under different experiment management, sequencing platform and target regions. **a**, observed species richness; **b**, Shannon's diversity index; **c**, Pielou's evenness; and **d**, Faith's phylogenetic diversity. All dots represent the percentage change in effect size between rhizosphere and bulk soils bacterial diversity at 95% confidence intervals (Cis). Mean values < 0 denote a higher diversity in the bacterial community of bulk soils (yellow dots; depletion in rhizosphere), whereas mean values > 0 reflect a significantly higher diversity in the rhizosphere bacterial community (blue dots). The intersection of error bars with the zero line indicates absence of significant difference between bacterial communities in rhizosphere and bulk soils (open dots). Sample size is showed by number of data pairs for each group. Experiment management: field and greenhouse; Sequencing platform: Illumina and Ion S5; Target regions: Bacterial V3~V4, V4~V5.

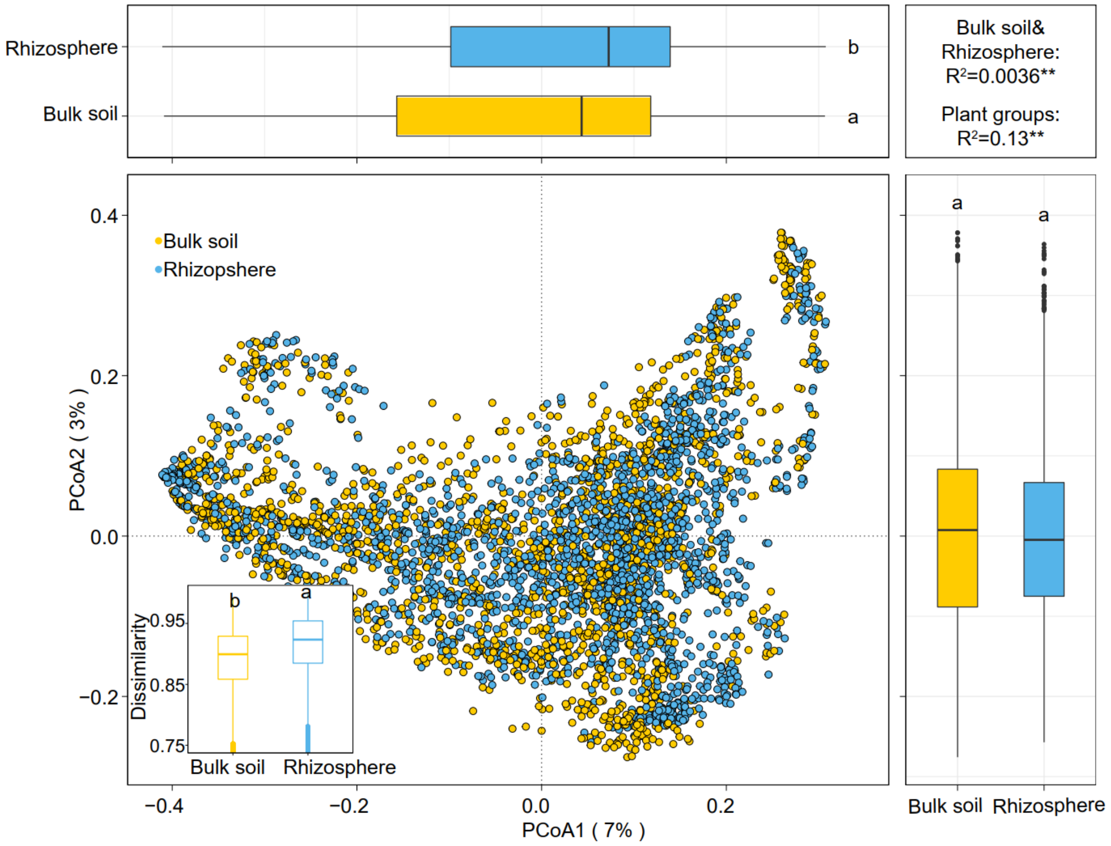

Supplementary Figure 4. Bacterial community composition of bulk soils and rhizosphere. Principal coordinate analysis (PcoA) plots depict the Bray-Curtis distance of the bacterial communities of the bulk soils and rhizosphere (bulk soils n = 1759 vs. rhizosphere n = 2182). Results from the PERMANOVA statistics tests testing the effect of the compartments (i.e., bulk soil and rhizosphere (**: p=0.01)) and the plant groups (**: p=0.01). R^2, explained variances, are provided on the top right of panel included on the plots. Box plots on the right and top show the overall distribution of PC1 and PC2 scores within each compartment. Boxplots reflect median (center line), 25th and 75th percentile values (bounds of boxes), and ranges (whiskers) for each category. Small letters indicate significant differences between bulk soils and rhizosphere (Tukey's test for multiple comparisons). Box plots at bottom right showed the beta dissimilarity via Bray-Curtis distance between samples within the same compartment. Statistical comparison was tested using two-sided Wilcoxon's rank sum tests.

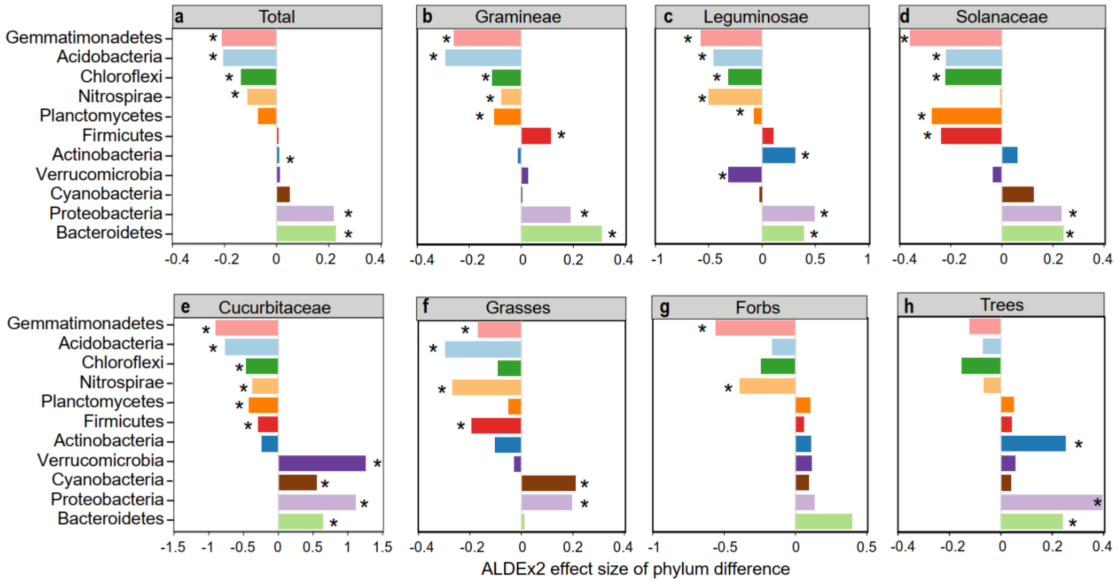

Supplementary Figure 5. The phyla differences between bacterial communities in rhizosphere and bulk soils depending on plant groups. **a**, total sample; **b**, Gramineae; **c**, Leguminosae; **d**, Solanaceae; **e**, Cucurbitaceae; **f**, grasses; **g**, forbs; **h**, trees. Positive values indicate higher relative abundance of the phylum in rhizosphere, while negative values indicate higher relative abundance of the phylum in the bulk soils. Statistical analysis performed with ALDEx2. Asterisk (*) indicated significantly differentially phylum with a false discovery rate (FDR)<0.05.

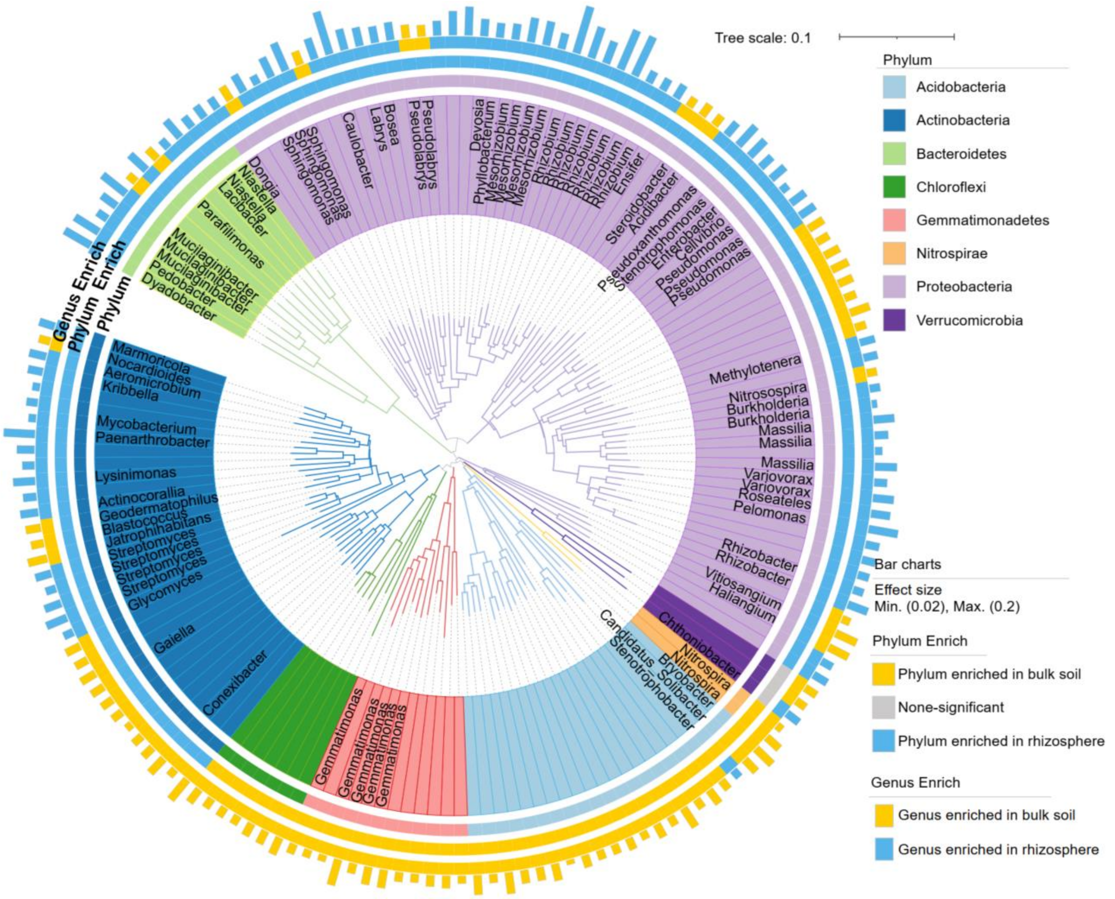

Supplementary Figure 6. Phylogeny of microorganisms with significant differences in bulk soils and rhizosphere bacterial communities. Phylogenetic tree comprising the 164 significant differences bacteria are labelled according to the genus name (exclude the unsigned genus) and the corresponding phylum is depicted in the first outer layer. The second outer layer showed the phylum enrichment between bulk soils and rhizosphere. The third outer layer represent the genus enrichment. The blue and yellow outer layer represent the ASVs with significant enrichment in the rhizosphere and bulk soils respectively. The outermost bar represents the ALDEx2 effect size between bulk soils and rhizosphere.

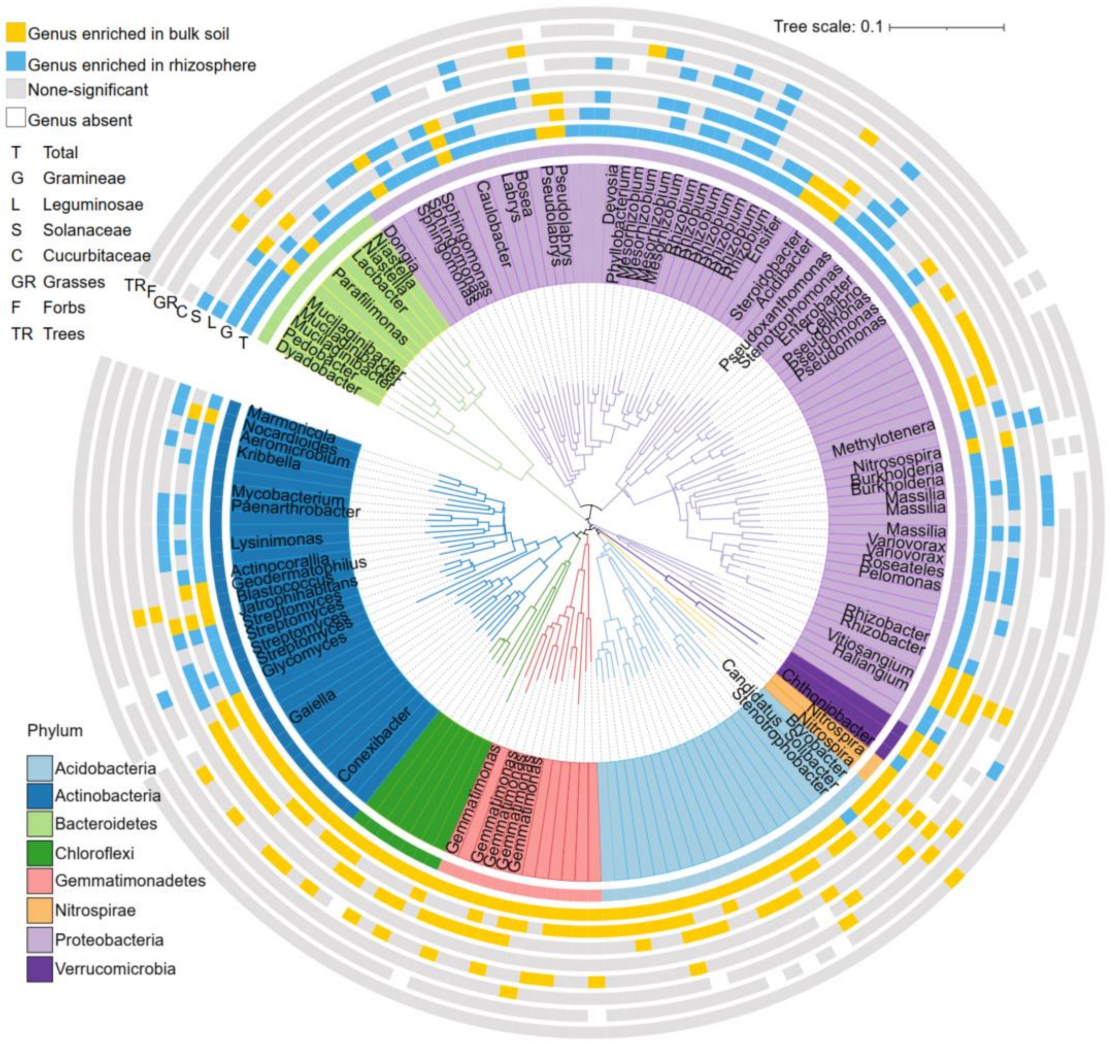

Supplementary Figure 7. Phylogeny of microorganisms with significant differences in bulk soils and rhizosphere bacterial communities depending on plant groups. The genus enriched in total samples (T) is depicted in the first outer layer, and then in turn Gramineae (G), Leguminosae (L), Solanaceae (S), Cucurbitaceae (C), grasses (GR), forbs (F), and trees (TR). The blue and yellow outer layer represent the ASVs with significant enrichment in the rhizosphere and bulk soils, respectively.

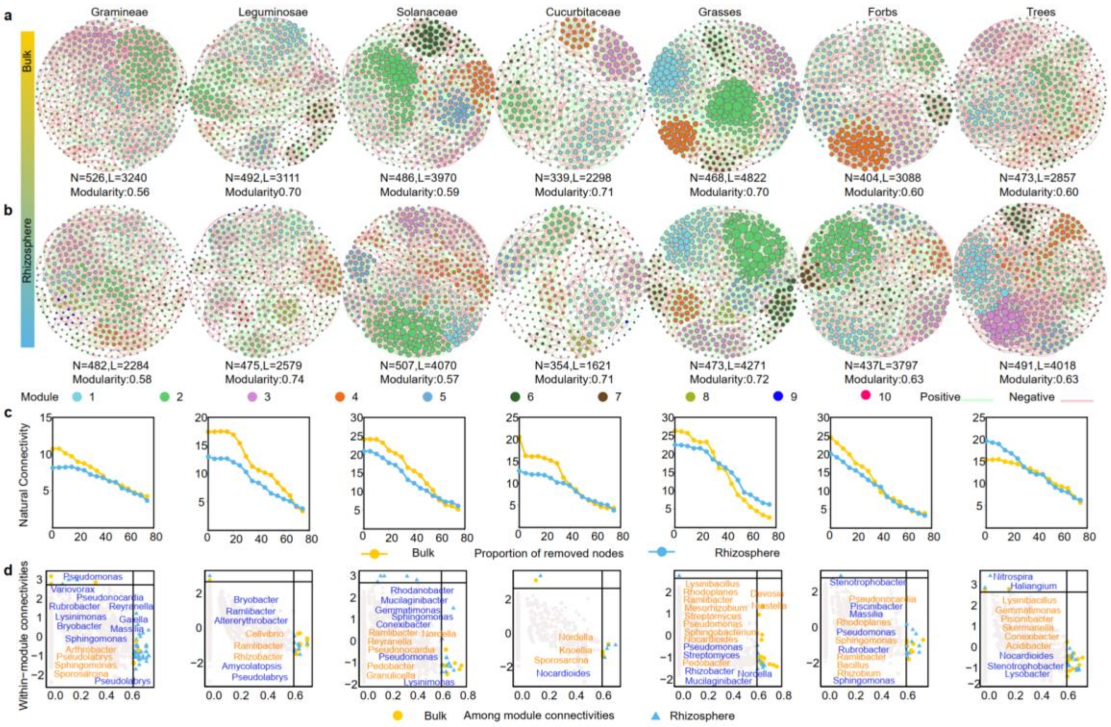

Supplementary Figure 8. Co-occurrence networks of bacterial ASVs in bulk soils and rhizosphere depending on plant groups. **a**, co-occurrence in bulk soils; **b**, co-occurrence in rhizosphere. Colors of nodes indicate the network modules. All of these networks come from Gramineae (bulk soils n = 725 vs. rhizosphere n = 847), Leguminosae (bulk soils n = 192 vs. rhizosphere n = 211), Solanaceae (bulk soils n = 134 vs. rhizosphere n = 173), Cucurbitaceae (bulk soils n = 28 vs. rhizosphere n = 56), grasses (bulk soils n = 163 vs. rhizosphere n = 191), forbs (bulk soils n = 27 vs. rhizosphere n = 66) and trees (bulk soils n = 137 vs. rhizosphere n = 161). **c**, robustness of bacterial network in bulk soils (yellow dots) and rhizosphere (blue dots); **d**, classification of nodes to identify keystone species within the bulk soils network and rhizosphere network.

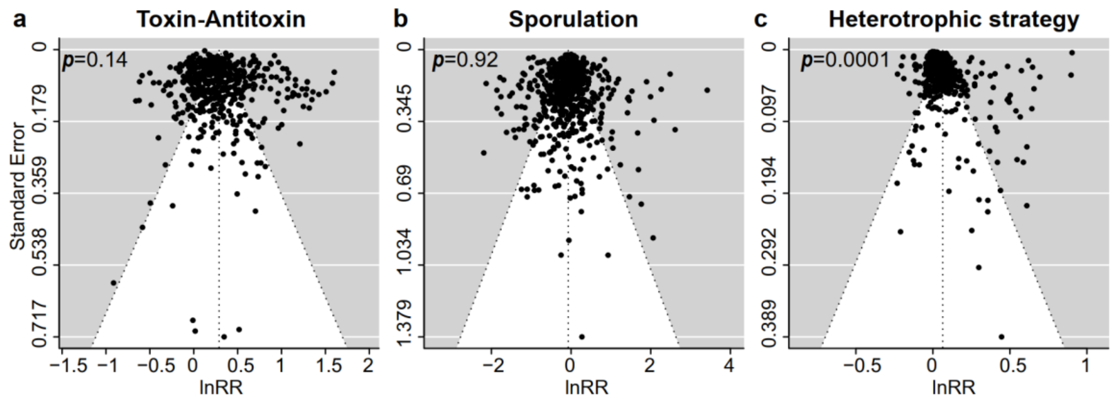

Supplementary Figure 9. Funnel plots for dormancy potentials and heterotrophic strategies of bacterial communities in rhizosphere and bulk soils. a, Funnel plot of toxin-antitoxin systems genes; **b**, Funnel plot of sporulation; **c**, Funnel plot of weighted mean ribosomal operon copy numbers. The top of each panel contains the results of publication bias tests using Egger's regression. A two-sided *p* value indicating statistical significance of the funnel plot asymmetry test. Asymmetry test showed a symmetric distribution ($p>0.05$) in toxin-antitoxin (a) and sporulation (b) while an asymmetric distribution ($p<0.05$) in weighted mean ribosomal operon copy numbers (c). lnRR: natural log-transformed response ratio.

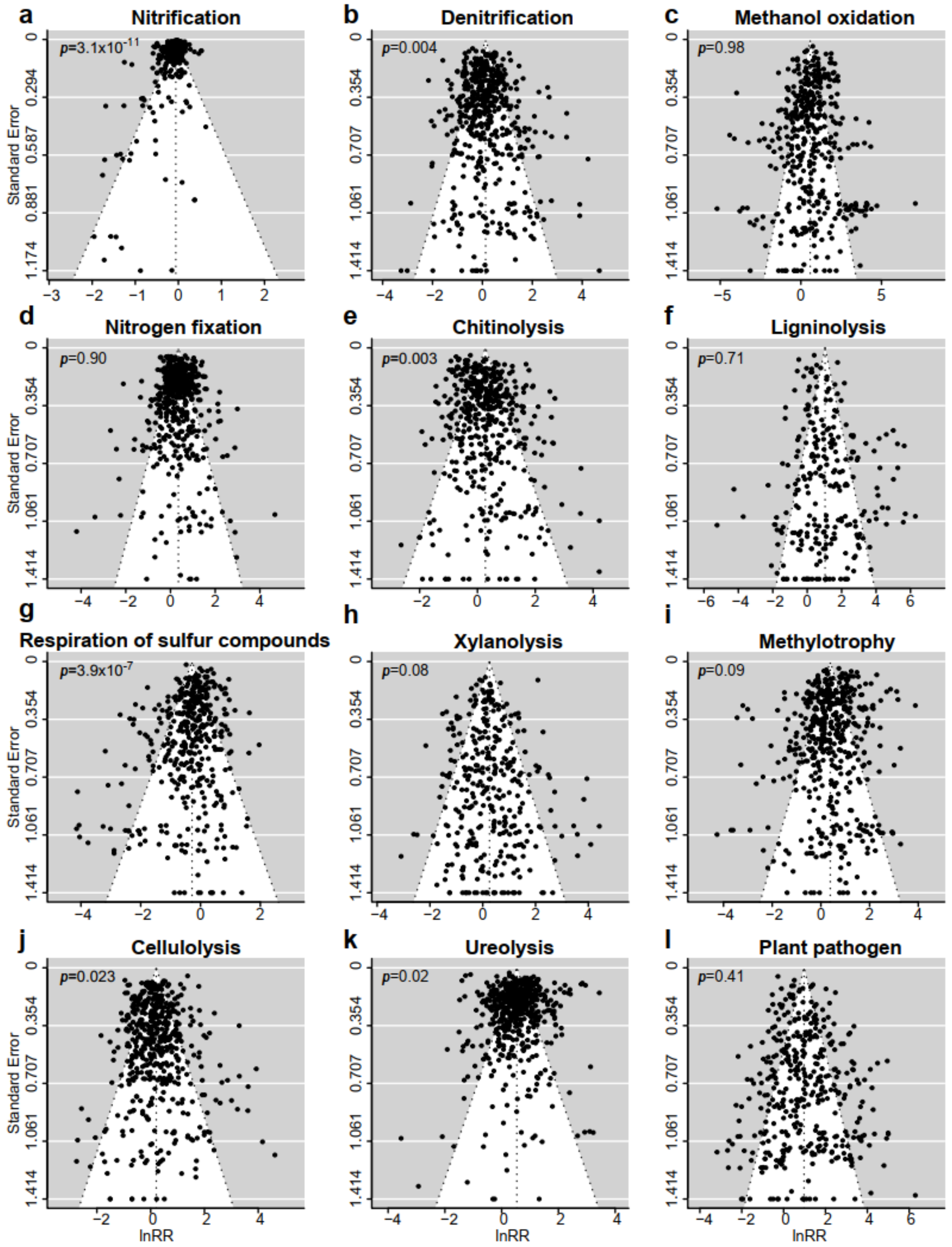

Supplementary Figure 10. Funnel plots for the functional potentials of bacterial communities in rhizosphere and bulk soils. a to **i**: nitrification, denitrification, methanol oxidation, nitrogen fixation, chitinolysis, ligninolysis, respiration of sulfur compounds, xylanolysis, methylotrophy, cellulolysis, ureolysis and plant pathogen. The top of each panel contains the results of publication bias tests using Egger's regression. A two-sided p value indicating statistical significance of the funnel plot asymmetry test. Asymmetry test showed a symmetric distribution (p>0.05) in methanol oxidation, nitrogen fixation, ligninolysis, xylanolysis, methylotrophy and plant pathogen and an asymmetric distribution (p<0.05) in nitrification, denitrification, chitinolysis, respiration of sulfur compounds, cellulolysis and ureolysis. lnRR: natural log-transformed response ratio.

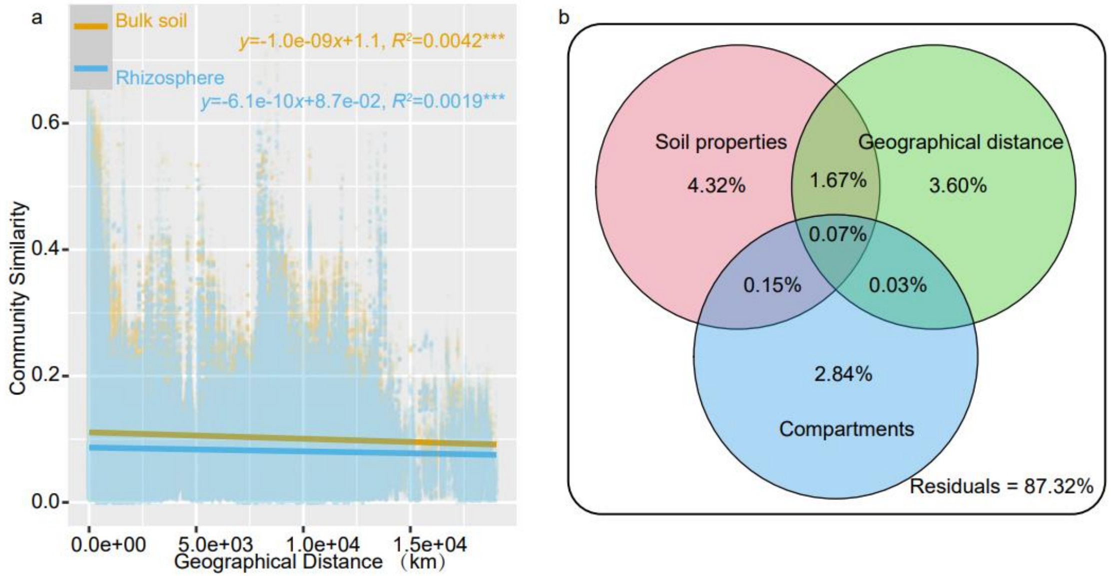

Supplementary Figure 11. Relationship between bacterial community similarity and geographical distance as well as the contributions of factors to bacterial community variation. **a**, Relationship between geographic distance and bacterial community similarity (based on Bray–Curtis distances) in bulk soils and rhizosphere. The solid yellow line and solid blue line represent the fitted linear regression model in bulk soil and rhizosphere, respectively. Asterisks (***) indicate the significance at $p < 0.001$ for the regression. **b**, Variance partition analysis shows the relative contributions of geographical distance, soil properties and compartments (bulk soil vs. rhizosphere) to the community variations. The explanations were statistically significant at $p < 0.05$ presented in the Venn diagram.

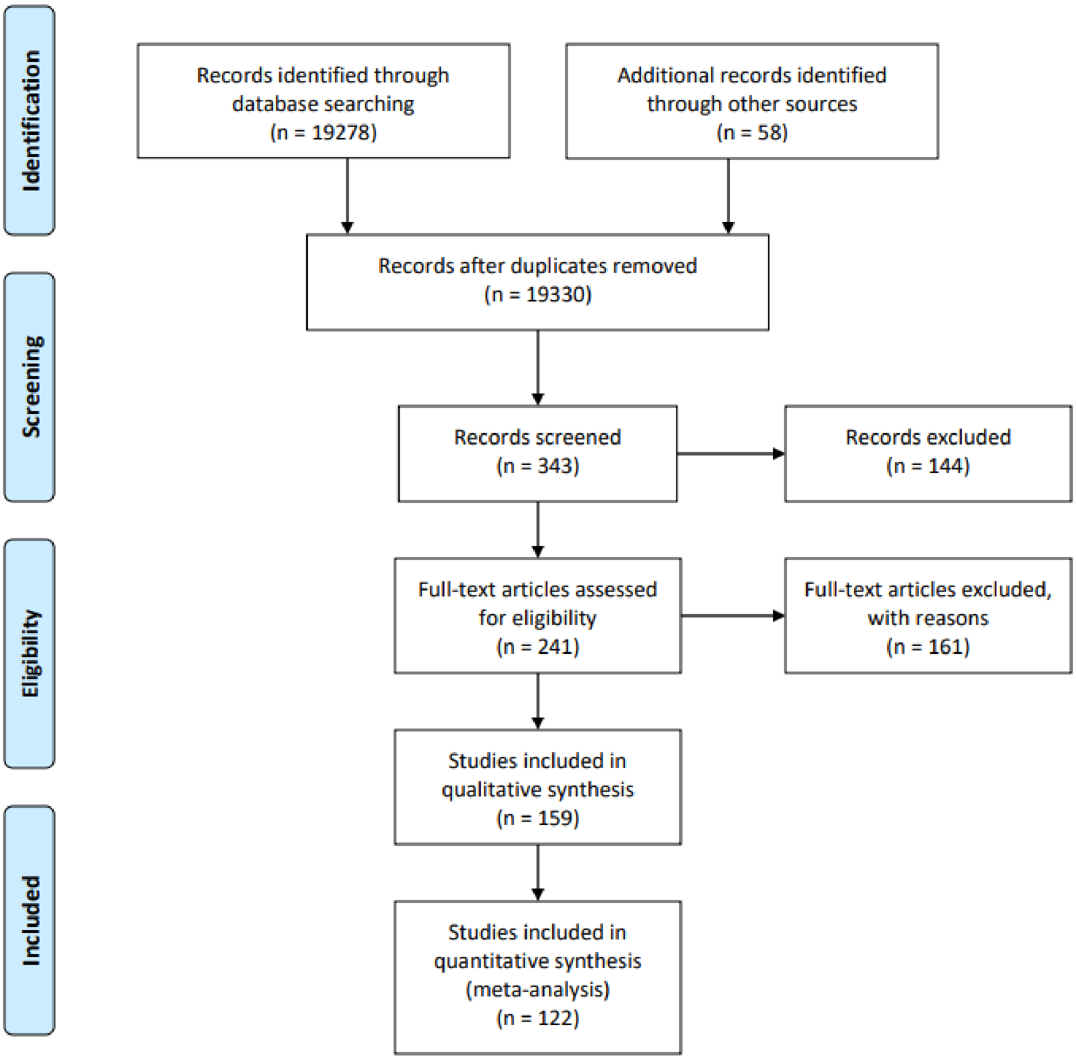

Supplementary Figure 12. PRISMA flow diagram for the studies selected and included in the systematic review.

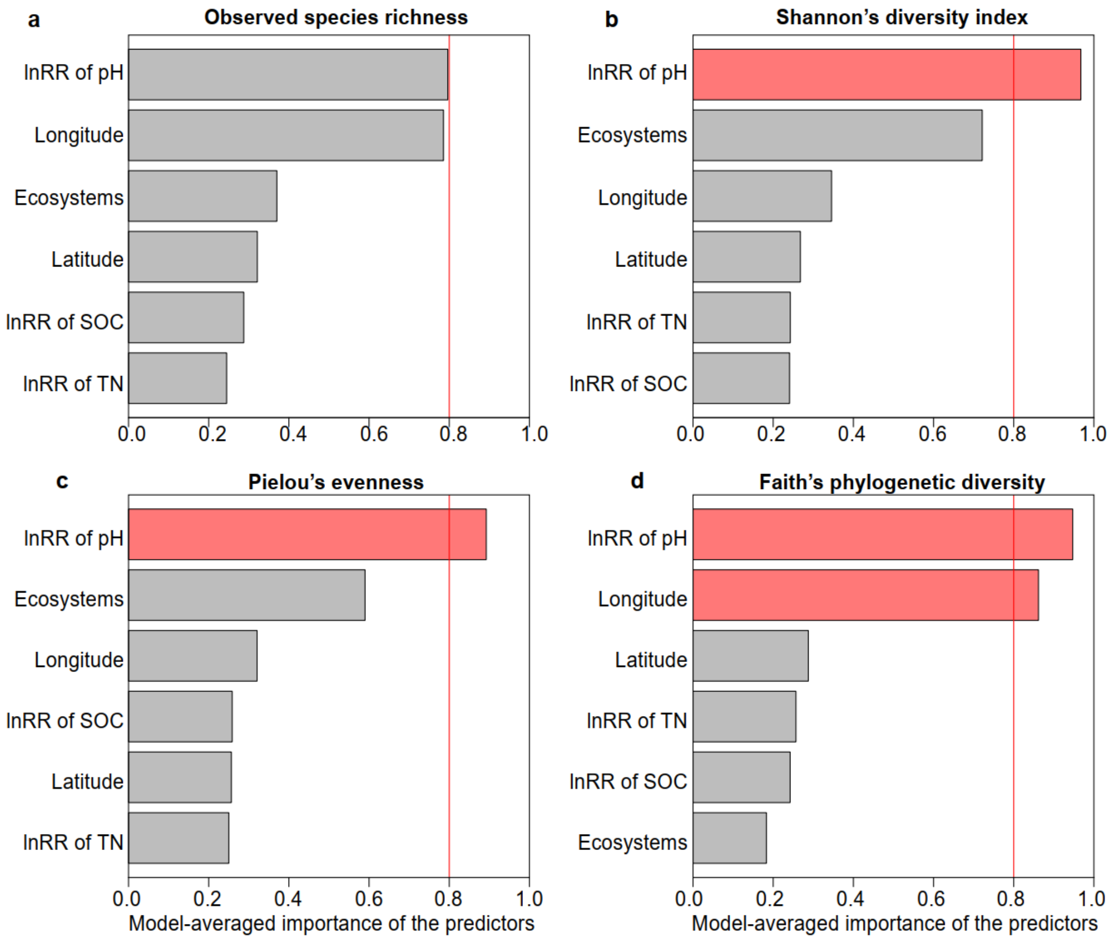

Supplementary Figure 13. Model-averaged importance of the predictors for natural log-transformed response ratios of bacterial diversity between bulk soils and rhizosphere. a, observed species richness; **b**, Shannon's diversity index; **c**, Pielou's evenness; and **d**, Faith's phylogenetic diversity. The importance value is estimated from the sum of Akaike weights derived from the model selection using corrected Akaike's information criteria. A cutoff of 0.8 (the red line) is set to determine the most important predictors. lnRR of pH: natural log-transformed response ratio of pH; lnRR of SOC: natural log-transformed response ratio of SOC; lnRR of TN: natural log-transformed response ratios of TN. The model selection analysis confirmed that natural log-transformed response ratios of bacterial Shannon's diversity index, Pielou's evenness and Faith's phylogenetic diversity were best predicted by changes in soil pH.

List of publications used in this synthesis.

1. Xia, Q., Rufty, T., Shi, W. Predominant Microbial Colonizers in the Root Endosphere and Rhizosphere of Turfgrass Systems: Pseudomonas veronii, Janthinobacterium lividum, and Pseudogymnoascus spp. *Front. Microbiol.* **12**, (2021).

2. Shi, Y., *et al.* Assembly of rhizosphere microbial communities in Artemisia annua: recruitment of plant growth-promoting microorganisms and inter-kingdom interactions between bacteria and fungi. *Plant Soil* (2021).

3. Pan, X., *et al.* Changes in the diversity and abundance of syntrophic and methanogenic communities in response to rice phenology. *Appl. Soil Ecol.* **159**, (2021).

4. Liu, Y., *et al.* Stochastic processes shape the biogeographic variations in core bacterial communities between aerial and belowground compartments of common bean. *Environ. Microbiol.* **23**, 949-964 (2021).

5. Song, L., *et al.* Characterization and comparison of the bacterial communities of rhizosphere and bulk soils from cadmium-polluted wheat fields. *Peerj* **8**, (2020).

6. Xiong, C., *et al.* Host selection shapes crop microbiome assembly and network complexity. *New Phytol.* **229**, 1091-1104 (2021).

7. Fan, K., Delgado-Baquerizo, M., Zhu, Y.-g., Chu, H. Crop production correlates with soil multitrophic communities at the large spatial scale. *Soil Biol. Biochem.* **151**, (2020).

8. Hernandez, E. G., Baraza, E., Smit, C., Berg, M. P., Salles, J. F. Salt Marsh Elevation Drives Root Microbial Composition of the Native Invasive Grass Elytrigia atherica. *Microorganisms* **8**, (2020).

9. Gagnon, V., *et al.* Life in mine tailings: microbial population structure across the bulk soil, rhizosphere, and roots of boreal species colonizing mine tailings in northwestern Quebec. *Ann. Microbiol.* **70**, (2020).

10. Taffner, J., Laggner, O., Wolfgang, A., Coyne, D., Berg, G. Exploring the Microbiota of East African Indigenous Leafy Greens for Plant Growth, Health, and Resilience. *Front. Microbiol.* **11**, (2020).

11. Jiao, Y., Chu, G., Yang, Z. a., Wang, Y., Wang, M. Bacterial Diversity in the Rhizosphere of Anabasis aphylla in the Gurbantunggut Desert, China. *Curr. Microbi.* **77**, 3750-3759 (2020).

12. Illescas, M., *et al.* Effect of Inorganic N Top Dressing and Trichoderma harzianum Seed-Inoculation on Crop Yield and the Shaping of Root Microbial Communities of Wheat Plants Cultivated Under High Basal N Fertilization. *Front. Plant Sci.* **11**, (2020).

13. Pinho, D., *et al.* Linking Tree Health, Rhizosphere Physicochemical Properties, and Microbiome in Acute Oak Decline. *Forests* **11**, (2020).

14. Zhuang, W., *et al.* Diversity, function and assembly of mangrove root-associated microbial communities at a continuous fine-scale. *Npj Biofilms Microbiomes* **6**, (2020).

15. Kinnunen-Grubb, M., Sapkota, R., Vignola, M., Nunes, I. M., Nicolaisen, M. Breeding selection imposed a differential selective pressure on the wheat root-associated microbiome. *FEMS Microbiol. Ecol.* **96**, (2020).

16. Ren, C., *et al.* Contrasting patterns of microbial community and enzyme activity between rhizosphere and bulk soil along an elevation gradient. *Catena* **196**,(2021).

17. Chen, L., Saixi, Y., Yi, R., Baoyin, T. Characterization of soil microbes associated with a grazing-tolerant grass species,Stipa breviflora, in the Inner Mongolian desert steppe. *Ecol. Evol.* **10**, 10607-10618 (2020).

18. Estendorfer, J., *et al.* Definition of Core Bacterial Taxa in Different Root Compartments of Dactylis glomerata, Grown in Soil under Different Levels of Land Use Intensity. *Diversity-Basel* **12**, (2020).

19. Zhang, Y., *et al.* Microbial communities in the rhizosphere and the root of lettuce as affected by Salmonella-contaminated irrigation water. *FEMS Microbiol. Ecol.* **94**, (2018).

20. Lu, G.-H., *et al.* Effects of an EPSPS-transgenic soybean line ZUTS31 on root-associated bacterial communities during field growth. *Plos One* **13**, (2018).

21. O'Brien, F. J. M., Dumont, M. G., Webb, J. S., Poppy, G. M. Rhizosphere Bacterial Communities Differ According to Fertilizer Regimes and Cabbage (Brassica oleracea var. capitata L.) Harvest Time, but Not Aphid Herbivory. *Front. Microbiol.* **9**, (2018).

22. Wu, Z., *et al.* Environmental factors shaping the diversity of bacterial communities that promote rice production. *Bmc Microbiol.* **18**, (2018).

23. Wang, Q., *et al.* Long-term fertilization changes bacterial diversity and bacterial communities in the maize rhizosphere of Chinese Mollisols. *Appl. Soil Ecol.* **125**, 88-96 (2018).

24. Fan, M., *et al.* Enhanced phytoremdiation of Robinia pseudoacacia in heavy metal-contaminated soils with rhizobia and the associated bacterial community structure and function. *Chemosphere* **197**, 729-740 (2018).

25. Cregger, M. A., *et al.* The Populus holobiont: dissecting the effects of plant niches and genotype on the microbiome. *Microbiome* **6**, 31 (2018).

26. Bowsher, A. W., Kearns, P. J., Popovic, D., Lowry, D. B., Shade, A. Locally Adapted Mimulus Ecotypes Differentially Impact Rhizosphere Bacterial and Archaeal Communities in an Environment-Dependent Manner. *Phytobiomes J.* **4**, 53-63 (2020).

27. Liu, H., *et al.* Utilization of marigold (Tagetes erecta) flower fermentation wastewater as a fertilizer and its effect on microbial community structure in maize rhizosphere and non-rhizosphere soil. *Biotechnol. Biotechnol. Equip.* **34**, 522-531 (2020).

28. Nahar, K., Floc'h, J.-B., Goyer, C., Zebarth, B. J., Whitney, S. Diversity of Soil Bacterial Community Is Influenced by Spatial Location and Time but Not Potato Cultivar. *Phytobiomes J.* **4**, 225-238 (2020).

29. Pepe-Ranney, C., Keyser, C., Trimble, J., Bissinger, B. Surveying the Sweetpotato Rhizosphere, Endophyte, and Surrounding Soil Microbiomes at Two North Carolina Farms Reveals Underpinnings of Sweetpotato Microbiome Community Assembly. *Phytobiomes J.* **4**: 75-89 (2020),.

30. Ruan, R., Jiang, Z., Wu, Y., Xu, M., Ni, J. High-throughput sequence analysis reveals variation in the relative abundance of components of the bacterial and fungal microbiota in the rhizosphere of Ginkgo biloba. *Peerj* **7**, (2019).

31. Li, L., *et al.* Dynamics and potential roles of abundant and rare subcommunities in the bioremediation of cadmium-contaminated paddy soil by Pseudomonas chenduensis. *Appl. Microbiol. Biotechnol.* **103**, 8203-8214 (2019).

32. Shen, Z., *et al.* Lime and ammonium carbonate fumigation coupled with bio-organic fertilizer application steered banana rhizosphere to assemble a unique microbiome against Panama disease. *Microb. Biotechnol.* **12**, 515-527 (2019).

33. Yamamoto, K., *et al.* Bacterial Diversity Associated With the Rhizosphere and Endosphere of Two Halophytes: Glaux maritima and Salicornia europaea. *Front. Microbiol.* **9**, 2878 (2018).

34. Praeg, N., Pauli, H., Illmer, P. Microbial Diversity in Bulk and Rhizosphere Soil of Ranunculus glacialis Along a High-Alpine Altitudinal Gradient. *Front. Microbiol.* **10**,(2019).

35. Cai, M., *et al.* Selenium induces changes of rhizosphere bacterial characteristics and enzyme activities affecting chromium/selenium uptake by pak choi (Brassica campestris L. ssp. Chinensis Makino) in chromium contaminated soil. *Environ. Pollut.* **249**, 716-727 (2019).

36. Kudjordjie, E. N., Sapkota, R., Steffensen, S. K., Fomsgaard, I. S., Nicolaisen, M. Maize synthesized benzoxazinoids affect the host associated microbiome. *Microbiome* **7**, (2019).

37. Lee, S. A., *et al.* A preliminary examination of bacterial, archaeal, and fungal communities inhabiting different rhizocompartments of tomato plants under real-world environments. *Sci.*

Rep. **9**, (2019).

38. Li, H., *et al.* Enrichment of phosphate solubilizing bacteria during late developmental stages of eggplant (Solanum melongena L.). *FEMS Microbiol. Ecol.* **95**, (2019).

39. Schoeps, R., *et al.* Land-Use Intensity Rather Than Plant Functional Identity Shapes Bacterial and Fungal Rhizosphere Communities. *Front. Microbiol.* **9**, (2018).

40. Singh, J., Silva, K. J. P., Fuchs, M., Khan, A. Potential role of weather, soil and plant microbial communities in rapid decline of apple trees. *Plos One* **14**, (2019).

41. Wen, Z.-L., *et al.* Enrichments/Derichments of Root-Associated Bacteria Related to Plant Growth and Nutrition Caused by the Growth of an EPSPS-Transgenic Maize Line in the Field. *Front. Microbiol.* **10**, (2019).

42. Wang, M., *et al.* Water management impacts the soil microbial communities and total arsenic and methylated arsenicals in rice grains. *Environ. Pollut.* **247**, 736-744 (2019).

43. Arafat, Y., *et al.* Soil Sickness in Aged Tea Plantation Is Associated With a Shift in Microbial Communities as a Result of Plant Polyphenol Accumulation in the Tea Gardens. *Front. Plant Sci.* **11**, (2020).

44. Balazs, H. E., Schmid, C. A. O., Podar, D., Hufnagel, G., Radl, V., Schroeder, P. Development of microbial communities in organochlorine pesticide contaminated soil: A post-reclamation perspective. *Appl. Soil Ecol.* **150**, (2020).

45. Bao, L., *et al.* Microbial community overlap between the phyllosphere and rhizosphere of three plants from Yongxing Island, South China Sea. *Microbiologyopen* **9**, (2020).

46. Bledsoe, R. B., Goodwillie, C., Peralta, A. L. Long-Term Nutrient Enrichment of an Oligotroph-Dominated Wetland Increases Bacterial Diversity in Bulk Soils and Plant Rhizospheres. *Msphere* **5**, (2020).

47. Chen, Y., *et al.* Miscanthus cultivation shapes rhizosphere microbial community structure and function as assessed by Illumina MiSeq sequencing combined with PICRUSt and FUNGUIld analyses. *Arch. Microbiol.* **202**, 1157-1171 (2020).

48. Chen, Z.-J., *et al.* Rhizosphere Bacterial Community Structure and Predicted Functional Analysis in the Water-Level Fluctuation Zone of the Danjiangkou Reservoir in China During the Dry Period. *Int. J. Environ. Res. Public Health* **17**, (2020).

49. Cheng, C., *et al.* Moss biocrusts buffer the negative effects of karst rocky desertification on soil properties and soil microbial richness. *Plant Soil* (2020).

50. Custer, G. F., van Diepen, L. T. A., Stump, W. L. Structural and Functional Dynamics of Soil Microbes following Spruce Beetle Infestation. *App. Environ. Microbiol.* **86**, (2020).

51. Dal Cortivo, C.*, et al.* Effects of Seed-Applied Biofertilizers on Rhizosphere Biodiversity and Growth of Common Wheat (Triticum aestivum L.) in the Field. *Front. Plant Sci.* **11**, (2020).

52. Deyett, E., Rolshausen, P. E. Endophytic microbial assemblage in grapevine. *FEMS Microbiol. Ecol.* **96**, (2020).

53. Fernandez-Gonzalez, A. J.*, et al.* Comparative study of neighboring Holm oak and olive trees-belowground microbial communities subjected to different soil management. *Plos One* **15**, (2020).

54. Garcia-Lemos, A. M.*, et al.* Under the Christmas Tree: Belowground Bacterial Associations With Abies nordmanniana Across Production Systems and Plant Development. *Front. Microbiol.* **11**, (2020).

55. Hernandez-Teran, A., Navarro-Diaz, M., Benitez, M., Lira, R., Wegier, A., Escalante, A. E. Host genotype explains rhizospheric microbial community composition: the case of wild cotton metapopulations (Gossypium hirsutum L.) in Mexico. *FEMS Microbiol. Ecol.* **96**, (2020).

56. Huang, C., Han, X., Yang, Z., Chen, Y., Rengel, Z. Sowing Methods Influence Soil Bacterial Diversity and Community Composition in a Winter Wheat-Summer Maize Rotation System on the Loess Plateau. *Front. Microbiol.* **11**, (2020).

57. Lei, H., Liu, A., Hou, Q., Zhao, Q., Guo, J., Wang, Z. Diversity patterns of soil microbial communities in theSophora flavescensrhizosphere in response to continuous monocropping. *Bmc Microbiol.* **20**, (2020).

58. Li, M., Yang, F., Wu, X., Yan, H., Liu, Y. Effects of continuous cropping of sugar beet (Beta vulgarisL.) on its endophytic and soil bacterial community by high-throughput sequencing. *Ann. Microbiol.* **70**, (2020).

59. Lopez-Lozano, N. E., Echeverria Molinar, A., Ortiz Duran, E. A., Hernandez Rosales, M., Souza, V. Bacterial Diversity and Interaction Networks ofAgave lechuguillaRhizosphere Differ Significantly From Bulk Soil in the Oligotrophic Basin of Cuatro Cienegas. *Front. Plant Sci.* **11**, (2020).

60. Lumibao, C. Y.*, et al.* Rhizosphere microbial communities reflect genotypic and trait variation in a salt marsh ecosystem engineer. *Am. J. Bot.* **107**, 941-949 (2020).

61. Mapelli, F., Riva, V., Vergani, L., Choukrallah, R., Borin, S. Unveiling the Microbiota Diversity of the XerophyteArgania spinosaL. Skeels Root System and Residuesphere. *Microb. Ecol.* **80**, 822-836 (2020).

62. Schneijderberg, M., *et al.* Quantitative comparison between the rhizosphere effect ofArabidopsis thalianaand co-occurring plant species with a longer life history. *ISME J.* **14**, 2433-2448 (2020).

63. Wang, P., Marsh, E. L., Kruger, G., Lorenz, A., Schachtman, D. P. Belowground microbial communities respond to water deficit and are shaped by decades of maize hybrid breeding. *Environ. Microbiol.* **22**, 889-904 (2020).

64. Yue, Y., *et al.* Microbiome structure and function in rhizosphere of Jerusalem artichoke grown in saline land. *Sci. Total Environ.* **724**, (2020).

65. Zhang, P., Cui, Z., Guo, M., Xi, R. Characteristics of the soil microbial community in the forestland of Camellia oleifera. *Peerj* **8**, (2020).

66. Zhang, W., *et al.* Short-Term Effects of Eco-Friendly Fertilizers on a Soil Bacterial Community in the Topsoil and Rhizosphere of an Irrigated Agroecosystem. *Sustainability* **12**, (2020).

67. Osman, J. R., Fernandes, G., DuBow, M. S. Bacterial diversity of the rhizosphere and nearby surface soil of rice (Oryza sativa) growing in the Camargue (France). *Rhizosphere* **3**, 112-122 (2017).

68. Lu, G. H., *et al.* Impact of a Glyphosate-Tolerant Soybean Line on the Rhizobacteria, Revealed by Illumina MiSeq. *J. Microbiol. Biotechnol.* **27**, 561-572 (2017).

69. da Costa, P. B., *et al.* Invasion ecology applied to inoculation of plant growth promoting bacteria through a novel SIMPER-PCA approach. *Plant Soil* **422**, 467-478 (2017).

70. Fernandez, A. L., Sheaffer, C. C., Wyse, D. L., Staley, C., Gould, T. J., Sadowsky, M. J. Structure of bacterial communities in soil following cover crop and organic fertilizer incorporation. *Appl. Microbiol.. Biotechnol.* **100**, 9331-9341 (2016).

71. Wang, P., Marsh, E. L., Ainsworth, E. A., Leakey, A. D. B., Sheflin, A. M., Schachtman, D. P. Shifts in microbial communities in soil, rhizosphere and roots of two major crop systems under elevated CO2 and O3. *Sci. Rep.* **7**, 15019 (2017).

72. Estendorfer, J., *et al.* The Influence of Land Use Intensity on the Plant-Associated Microbiome of Dactylis glomerata L. *Front. Plant Sci.* **8**, 930 (2017).

73. Liu, J., *et al.* Distinct soil bacterial communities in response to the cropping system in a Mollisol of northeast China. *Appl. Soil Ecol.* **119**, 407-416 (2017).

74. Moroenyane, I., Tremblay, J., Yergeau, E. Temporal and spatial interactions modulate the soybean microbiome. *FEMS Microbiol. Ecol.* **97**, (2021).

75. Zhou, Y., *et al.* Nitrifying Microbes in the Rhizosphere of Perennial Grasses Are Modified by Biological Nitrification Inhibition. *Microorganisms* **8**, (2020).

76. Brink, C. J., Postma, A., Slabbert, E., Postma, F., Muasya, A. M., Jacobs, K. Bacterial communities associated with natural and commercially grown rooibos (Aspalathus linearis). *Pedosphere* **30**, 778-790 (2020).

77. Wang, X., *et al.* An amplification-selection model for quantified rhizosphere microbiota assembly. *Science Bulletin.* **65**, 983-986 (2020).

78. Xu, Y., Ge, Y., Song, J., Rensing, C. Assembly of root-associated microbial community of typical rice cultivars in different soil types. *Biol. Fertil. Soils* **56**, 249-260 (2020).

79. Naylor, D., DeGraaf, S., Purdom, E., Coleman-Derr, D. Drought and host selection influence bacterial community dynamics in the grass root microbiome. *ISME J.* **11**, 2691-2704 (2017).

80. Xu, L., *et al.* Drought delays development of the sorghum root microbiome and enriches for monoderm bacteria. *Proc. Natl. Acad. Sci. USA* **115**, E4284-E4293 (2018).

81. Schlatter, D. C., Yin, C., Hulbert, S., Paulitz, T. C. Core Rhizosphere Microbiomes of Dryland Wheat Are Influenced by Location and Land Use History. *App. Environ. Microbiol.* **86**, (2020).

82. Tienda, S., *et al.* Soil Application of a Formulated Biocontrol Rhizobacterium,Pseudomonas chlororaphisPCL1606, Induces Soil Suppressiveness by Impacting Specific Microbial Communities. *Front. Microbiol.* **11**, (2020).

83. Sun, A., *et al.* Microbial communities in crop phyllosphere and root endosphere are more resistant than soil microbiota to fertilization. *Soil Biol. Biochem.* **153**, (2021).

84. Xu, H., Du, H., Zeng, F., Song, T., Peng, W. Diminished rhizosphere and bulk soil microbial abundance and diversity across succession stages in Karst area, southwest China. *Appl. Soil Ecol.* **158**, (2021).

85. Fan, K., *et al.* Rhizosphere-associated bacterial network structure and spatial distribution differ significantly from bulk soil in wheat crop fields. *Soil Biol. Biochem.* **113**, 275-284 (2017).

86. Chen, Y., Sun, R., Sun, T., Liang, Y., Jiang, Y., Sun, B. Organic amendments shift the phosphorus-correlated microbial co-occurrence pattern in the peanut rhizosphere network during long-term fertilization regimes. *Appl. Soil Ecol.* **124**, 229-239 (2018).

87. Kong, X., *et al.* Maize (Zea mays L. Sp.) varieties significantly influence bacterial and fungal community in bulk soil, rhizosphere soil and phyllosphere. *FEMS Microbiol. Ecol.* **96**, (2020).

88. Liu, Z., *et al.* Long-term continuous cropping of soybean is comparable to crop rotation in mediating microbial abundance, diversity and community composition. *Soil Tillage. Res.* **197**, (2020).

89. Yin, J., Yu, Y., Zhang, Z., Chen, L., Ruan, L. Enrichment of potentially beneficial bacteria from the consistent microbial community confers canker resistance on tomato. *Microbiol. Res.* **234**, 126446-126446 (2020).

90. Deng, X., *et al.* Rhizosphere bacteria assembly derived from fumigation and organic amendment triggers the direct and indirect suppression of tomato bacterial wilt disease. *Appl. Soil Ecol.* **147**, (2020).

91. Hu, X., *et al.* Dramatic changes in bacterial co-occurrence patterns and keystone taxa responses to cropping systems in Mollisols of Northeast China. *Arch. Agron. Soil Sci.* (2020).

92. Krause, S. M. B., Dohrmann, A. B., Gillor, O., Christensen, B. T., Merbach, I., Tebbe, C. C. Soil properties and habitats determine the response of bacterial communities to agricultural wastewater irrigation. *Pedosphere* **30**, 146-158 (2020).

93. Zhao, M., *et al.* Predominance of soil vs root effect in rhizosphere microbiota reassembly. *FEMS Microbiol. Ecol.* **95**, (2019).

94. Schmidt, J. E., Kent, A. D., Brisson, V. L., Gaudin, A. C. M. Agricultural management and plant selection interactively affect rhizosphere microbial community structure and nitrogen cycling. *Microbiome* **7**, (2019).

95. Kopecky, J., *et al.* Bacterial, archaeal and micro-eukaryotic communities characterize a disease-suppressive or conducive soil and a cultivar resistant or susceptible to common scab. *Sci.Rep.* **9**, (2019).

96. Cheng, J., Lee, X., Tang, Y., Zhang, Q. Long-term effects of biochar amendment on rhizosphere and bulk soil microbial communities in a karst region, southwest China. *Appl. Soil Ecol.* **140**, 126-134 (2019).

97. Liu, F., Hewezi, T., Lebeis, S. L., Pantalone, V., Grewal, P. S., Staton, M. E. Soil indigenous microbiome and plant genotypes cooperatively modify soybean rhizosphere microbiome assembly. *Bmc Microb.* **19**, (2019).

98. Kavamura, V. N., *et al.* Inorganic Nitrogen Application Affects Both Taxonomical and Predicted Functional Structure of Wheat Rhizosphere Bacterial Communities. *Front. Microbiol.* **9**, (2018).

99. Aguirre-von-Wobeser, E., Rocha-Estrada, J., Shapiro, L. R., de la Torre, M. Enrichment of Verrucomicrobia, Actinobacteria and Burkholderiales drives selection of bacterial community

from soil by maize roots in a traditional milpa agroecosystem. *Plos One* **13**, (2018).

100. Visioli, G., Sanangelantoni, A. M., Vamerali, T., Dal Cortivo, C., Blandino, M. 16S rDNA Profiling to Reveal the Influence of Seed-Applied Biostimulants on the Rhizosphere of Young Maize Plants. *Molecules* **23**, (2018).

101. Maarastawi, S. A., Frindte, K., Linnartz, M., Knief, C. Crop Rotation and Straw Application Impact Microbial Communities in Italian and Philippine Soils and the Rhizosphere of Zea mays. *Front. Microbiol.* **9**, (2018).

102. Granzow, S., *et al.* The Effects of Cropping Regimes on Fungal and Bacterial Communities of Wheat and Faba Bean in a Greenhouse Pot Experiment Differ between Plant Species and Compartment. *Front. Microbiol.* **8**, (2017).

103. Lopes, L. D., Pereira e Silva, M. d. C., Andreote, F. D. Bacterial Abilities and Adaptation Toward the Rhizosphere Colonization. *Front. Microbiol.* **7**, (2016).

104. Edwards, J., *et al.* Soil domestication by rice cultivation results in plant-soil feedback through shifts in soil microbiota. *Genome Biol.* **20**, 221 (2019).

105. Walters, W. A., *et al.* Large-scale replicated field study of maize rhizosphere identifies heritable microbes. *Proc. Natl. Acad. Sci. USA* **115**, 7368-7373 (2018).

106. Jin, T., *et al.* Taxonomic structure and functional association of foxtail millet root microbiome. *Gigascience* **6**, 1-12 (2017).

107. Zhou, J., *et al.* Comparison of bacterial and archaeal communities in two fertilizer doses and soil compartments under continuous cultivation system of garlic. *Plos One* **16**, (2021).

108. Zhou, J., Fong, J. J. Strong agricultural management effects on soil microbial community in a non-experimental agroecosystem. *Appl. Soil Ecol.* **165**, (2021).

109. Yan, L., Zhang, W., Duan, W., Zhang, Y., Zheng, W., Lai, X. Temporal Bacterial Community Diversity in the Nicotiana tabacum Rhizosphere Over Years of Continuous Monocropping. *Front. Microbiol.* **12**, (2021).

110. Shao, J., *et al.* Rhizosphere microbiome assembly involves seed-borne bacteria in compensatory phosphate solubilization. *Soil Biol. Biochem.* **159**, (2021).

111. Bettermann, A., *et al.* Importance of microbial communities at the root-soil interface for extracellular polymeric substances and soil aggregation in semiarid grasslands. *Soil Biol. Biochem.* **159**, (2021).

112. Li, W., *et al.* Structure and driving factors of the soil microbial community associated with

Alhagi sparsifolia in an arid desert. *Plos One* **16**, (2021).

113. Cao, H., Gao, G., Zhang, Y., Guo, M., Ren, Y., Ding, G. Soil bacterial approach to assessing afforestation in the desertfied Northern China. *J. Clean. Prod.* **292**, (2021).

114. Liu, L., Huang, X., Zhang, J., Cai, Z., Jiang, K., Chang, Y. Deciphering the relative importance of soil and plant traits on the development of rhizosphere microbial communities. *Soil Biol. Biochem.* **148**, (2020).

115. Zhao, J., *et al.* Manipulation of the rhizosphere microbial community through application of a new bio-organic fertilizer improves watermelon quality and health. *PLoS One* **13**, e0192967 (2018).

116. McKnight, M. M., Grogan, P., Walker, V. K. Impact of long-term fertilizer and summer warming treatments on bulk soil and birch rhizosphere microbial communities in mesic arctic tundra. *Arct. Antarct. Alp. Res.* **53**, 196-211 (2021).

117. Sun, W., *et al.* Paddy soil microbial communities driven by environment- and microbe-microbe interactions: A case study of elevation-resolved microbial communities in a rice terrace. *Sci. Total Environ.* **612**, 884-893 (2018).

118. Li, S., *et al.* Short-term lime application impacts microbial community composition and potential function in an acid black soil. *Plant Soil* (2021).

119. Chou, M.-Y., Shrestha, S., Rioux, R., Koch, P. Hyperlocal Variation in Soil Iron and the Rhizosphere Bacterial Community Determines Dollar Spot Development in Amenity Turfgrass. *App. Environ. Microbiol.* **87**, (2021).

120. Habiyaremye, J. D. D., Goldmann, K., Reitz, T., Herrmann, S., Buscot, F. Tree Root Zone Microbiome: Exploring the Magnitude of Environmental Conditions and Host Tree Impact. *Front. Microbiol.* **11**, (2020).

121. Wu, N., Shi, W., Liu, W., Gao, Z., Han, L., Wang, X. Differential impact of Bt-transgenic rice plantings on bacterial community in three niches over consecutive years. *Ecotoxicol. Environ. Saf.* **223**, 112569 (2021).

122. Hu, X., *et al.* Conventional and conservation tillage practices affect soil microbial co-occurrence patterns and are associated with crop yields. *Agric. Ecosyst. Environ.* **319**, (2021).
