# Supplementary material for: Rhizosphere bacteriome structure and functions
Source: Nat Commun. 2022 Feb 11;13:836. doi: 10.1038/s41467-022-28448-9 (PMC8837802; doi:10.1038/s41467-022-28448-9)
Supplement: Supplementary file 2 — Reporting Summary [file 41467_2022_28448_MOESM2_ESM.pdf]

nature research

Corresponding author(s): Ning Ling, Yakov Kuzyakov

Last updated by author(s): Jan 23, 2022

# Reporting Summary

Nature Research wishes to improve the reproducibility of the work that we publish. This form provides structure for consistency and transparency in reporting. For further information on Nature Research policies, see our Editorial Policies and the Editorial Policy Checklist.

## Statistics

For all statistical analyses, confirm that the following items are present in the figure legend, table legend, main text, or Methods section.

| n/a | Confirmed | |
|---|---|---|
| ☐ | ☒ | The exact sample size ($n$) for each experimental group/condition, given as a discrete number and unit of measurement |
| ☒ | ☐ | A statement on whether measurements were taken from distinct samples or whether the same sample was measured repeatedly |
| ☐ | ☒ | The statistical test(s) used AND whether they are one- or two-sided<br>*Only common tests should be described solely by name; describe more complex techniques in the Methods section.* |
| ☐ | ☒ | A description of all covariates tested |
| ☐ | ☒ | A description of any assumptions or corrections, such as tests of normality and adjustment for multiple comparisons |
| ☐ | ☒ | A full description of the statistical parameters including central tendency (e.g. means) or other basic estimates (e.g. regression coefficient) AND variation (e.g. standard deviation) or associated estimates of uncertainty (e.g. confidence intervals) |
| ☐ | ☒ | For null hypothesis testing, the test statistic (e.g. $F$, $t$, $r$) with confidence intervals, effect sizes, degrees of freedom and $P$ value noted<br>*Give P values as exact values whenever suitable.* |
| ☒ | ☐ | For Bayesian analysis, information on the choice of priors and Markov chain Monte Carlo settings |
| ☒ | ☐ | For hierarchical and complex designs, identification of the appropriate level for tests and full reporting of outcomes |
| ☐ | ☒ | Estimates of effect sizes (e.g. Cohen's $d$, Pearson's $r$), indicating how they were calculated |

*Our web collection on statistics for biologists contains articles on many of the points above.*

## Software and code

Policy information about availability of computer code

| | |
|---|---|
| Data collection | Office Microsoft Excel 2016 and NCBI SRA Toolkit (version 2.9.2) were used for data collection. |
| Data analysis | Raw paired-end sequences were analyzed in Qiime2 software, and then some software, including MetaWin (Version 2.1.4), R software (version 3.6.0, R Core Team, 2019), Treemap (Version 4.1.1) software, Cytoscape 3.7.2 software and Gephi 0.9.2 software were employed in downstream analysis. The code for analyses can be found under https://github.com/wangtingting0104/Data-analysis |

For manuscripts utilizing custom algorithms or software that are central to the research but not yet described in published literature, software must be made available to editors and reviewers. We strongly encourage code deposition in a community repository (e.g. GitHub). See the Nature Research guidelines for submitting code & software for further information.

## Data

Policy information about availability of data

All manuscripts must include a data availability statement. This statement should provide the following information, where applicable:

- Accession codes, unique identifiers, or web links for publicly available datasets
- A list of figures that have associated raw data
- A description of any restrictions on data availability

The authors declare that all published data will be available within its Supplementary information when publishing. The source data underlying the main figures are provided as a Source Data file. The sequences obtained in this study are previously published and publicly available sequences that were downloaded from the NCBI Sequence Read Archive (https://www.ncbi.nlm.nih.gov/).

# Field-specific reporting

Please select the one below that is the best fit for your research. If you are not sure, read the appropriate sections before making your selection.

- [ ] Life sciences
- [ ] Behavioural & social sciences
- [x] Ecological, evolutionary & environmental sciences

For a reference copy of the document with all sections, see nature.com/documents/nr-reporting-summary-flat.pdf

# Ecological, evolutionary & environmental sciences study design

All studies must disclose on these points even when the disclosure is negative.

| | |
|---|---|
| Study description | We generalized bacterial traits regarding community diversity, composition and functions using published 16s rDNA amplicon sequences of 557 pairs of bulk soils vs rhizosphere of plants across ecosystems. |
| Research sample | We followed the PRISMA protocols for study selection and inclusion in the systematic meta-analysis. The peer-reviewed literature was firstly searched through Web of Science. Details about the inclusion/exclusion criteria for publications are reported in Methods. In total, 557 cases from 122 quantitative studies were identified, focusing on the effects of bacterial communities between bulk soils and rhizosphere. |
| Sampling strategy | With the aim of constructing a comprehensive database of the experimentally determined the differences of bacterial communities between bulk soils and rhizosphere, we tried to collected as many as experiments that fulfill our criteria (describe below) as possible. We followed the PRISMA protocol for study selection and inclusion. The peer-reviewed literature was firstly searched through ISI Web of Science. Details about the inclusion/exclusion criteria for publications are reported in Methods. |
| Data collection | TTW and colleagues in the research group conducted literature search and collected the data following the protocol designed jointly with NL. Literatures were initially assessed for relevance first by title, as well as keywords if there were available, then by abstract and finally by full text. We extracted mean, statistical variation (i.e., standard error, standard deviation) and sample size for bulk soil and rhizosphere samples. We also extracted the information of study site (i.e., country, coordinates of study area), plant species for each case. |
| Timing and spatial scale | The analyzed studies published between 2010 and July 2021, spanning a total of 20 countries and regions.Our data was classified into cropland, forest and grassland ecosystems. |
| Data exclusions | We excluded the data which did not correctly deposite in the NCBI database or the deposited data can not be recognized in their corresponding treatments or the downloaded data did not obey the rule of quality control. |
| Reproducibility | The methods of data collection and analysis are presented in the Methods section in detail and to enhance reproducibility. The raw data and code for each main figure was also provided as the Source Data. |
| Randomization | This research was based on observation from already published studies, the authors did not affect experimental design of individual studies. Meta-analysis is analysis of large datasets and randomization can be carried out for large datasets |
| Blinding | Blinding is not applicable for this study, because methods used in the assessment of the results were objective. Blinding is also not necessary because the results are quantitative and did not require subjective judgment or interpretation. Blinding is not typically used in the field. |

Did the study involve field work? [ ] Yes [x] No

# Reporting for specific materials, systems and methods

We require information from authors about some types of materials, experimental systems and methods used in many studies. Here, indicate whether each material, system or method listed is relevant to your study. If you are not sure if a list item applies to your research, read the appropriate section before selecting a response.

## Materials & experimental systems

| n/a | Involved in the study | |
|---|---|---|
| x | | Antibodies |
| x | | Eukaryotic cell lines |
| x | | Palaeontology and archaeology |
| x | | Animals and other organisms |
| x | | Human research participants |
| x | | Clinical data |
| x | | Dual use research of concern |

## Methods

| n/a | Involved in the study | |
|---|---|---|
| x | | ChIP-seq |
| x | | Flow cytometry |
| x | | MRI-based neuroimaging |
